# Does bilingualism come with linguistic costs? A meta-analytic review of the bilingual lexical deficit

**DOI:** 10.3758/s13423-022-02136-7

**Published:** 2022-11-03

**Authors:** Emanuel Bylund, Jan Antfolk, Niclas Abrahamsson, Anne Marte Haug Olstad, Gunnar Norrman, Minna Lehtonen

**Affiliations:** 1 Department of General Linguistics, Stellenbosch University, Stellenbosch, South Africa; 2 Stockholm University, Stockholm, Sweden; 3 Åbo Akademi University, Turku, Finland; 4 University of Oslo, Oslo, Norway; 5 University of Turku, Turku, Finland

**Keywords:** Age of acquisition, Bilingualism, Lexical deficit, Executive control, Vocabulary

## Abstract

A series of recent studies have shown that the once-assumed cognitive advantage of bilingualism finds little support in the evidence available to date. Surprisingly, however, the view that bilingualism incurs linguistic costs (the so-called lexical deficit) has not yet been subjected to the same degree of scrutiny, despite its centrality for our understanding of the human capacity for language. The current study implemented a comprehensive meta-analysis to address this gap. By analyzing 478 effect sizes from 130 studies on expressive vocabulary, we found that observed lexical deficits could not be attributed to bilingualism: Simultaneous bilinguals (who acquired both languages from birth) did not exhibit any lexical deficit, nor did sequential bilinguals (who acquired one language from birth and a second language after that) when tested in their mother tongue. Instead, systematic evidence for a lexical deficit was found among sequential bilinguals when tested in their second language, and more so for late than for early second language learners. This result suggests that a lexical deficit may be a phenomenon of second language acquisition rather than bilingualism per se.

**Supplementary Information:**

The online version contains supplementary material available at 10.3758/s13423-022-02136-7.

Does bilingualism cause any changes to the human mind? Up until recently, the standard answer to this question would have been that acquiring, mastering, and using two (or more) languages affords certain cognitive advantages, while also incurring certain linguistic costs. The advantages would be particularly salient in bilingual individuals’ enhanced cognitive flexibility and ability to monitor and control their actions and impulses (i.e., executive functioning). The costs would be manifested as a so-called lexical deficit, a term implying that bilinguals, relative to monolinguals, exhibit smaller vocabulary knowledge and slower word retrieval. For instance, in a picture-naming task, bilingual participants would be more likely to take longer to retrieve the words for the displayed items and more likely to name fewer items, than their monolingual counterparts. Similarly, in a verbal fluency task, bilinguals would be likely to name fewer category members (e.g., all the vegetables they can think of, or all the words beginning with the letter “p”) relative to monolingual participants.^1^

Recently, doubt has been cast upon the view that bilingualism affords a cognitive advantage. A number of studies have drawn attention to publication bias and inconsistencies in the findings on the bilingual advantage (e.g., de Bruin et al., 2015; Hilchey & Klein, 2011; Paap & Greenberg, 2013), and various large-scale investigations (e.g., Dick et al., 2019; Nichols et al., 2020) and comprehensive meta-analyses (Anderson et al., 2020; Donnelly et al., 2019; Lehtonen et al., 2018) have found either a small or no effect of bilingualism on cognitive functioning.

Surprisingly, the view that bilingualism entails a lexical deficit has, as of yet, not been subjected to nearly as much scrutiny. The current study addresses this gap by means of a comprehensive meta-analysis of the evidence available to date. Testing the extent and robustness of any detected lexical deficit is important for a number of reasons: First, at a more general level, knowledge about the potential effects of bilingualism on linguistic abilities is crucial for advancing our understanding of the human capacity for language (Bialystok & Werker, 2017). Second, recent findings show that certain verbal behaviours that have been characterized as a “cost” of bilingualism (e.g., diverging knowledge in phonology and grammar) are in fact a consequence of language learning history (e.g., age of language acquisition) and not of bilingualism per se (Bylund et al., 2019; Bylund et al., 2021; Hyltenstam et al., 2009; Norrman & Bylund, 2016; Veríssimo et al., 2018). Whether the same applies to the lexical deficit remains to be seen.

Looking closer at the bilingual lexical deficit also has relevance for evaluating current models on language development. Assuming principles of Hebbian learning at its core, one set of models predict that *amount of exposure* to a language is the driving mechanism behind word learning: In order for a given lexical item to be acquired and activated, a certain amount of exposure is necessary (e.g., Gollan et al., 2005b; Gollan et al., 2011). Because bilinguals have to divide the hours of the day between their two languages, it is impossible for them to receive the same amount of exposure to each language as monolingual individuals do to their one language. As a consequence, lexical representations in bilingualism are weaker and have higher activation thresholds. Another set of frameworks instead holds that it is the *timing of exposure* that primarily determines the strength of lexical representation (e.g., Hernandez et al., 2005; P. Li, 2009; Werker & Hensch, 2015). While recognizing the importance of exposure, these approaches hold that languages learnt after the mother tongue (L1)—even if learnt in early childhood—will be parasitic on L1 representations and, moreover, exhibit nonnativelike phonetic categories, which will ultimately compromise lexical development (Choi et al., 2018; Rivera-Gaxiola et al., 2005).

These approaches to lexical development differ with regards to their predictions of the existence of a lexical deficit. Whereas the amount-of-exposure models regard the lexical deficit as an integral part of bilingualism, the timing-of-exposure models, though not necessarily questioning the existence of a lexical deficit, are compatible with the idea that bilingualism per se need not incur linguistic costs: if both languages are acquired from birth, there will be no prior entrenched system that leads to lexical parasitism, and no compromised phonetic categories.

The following three alternative hypotheses may be formulated:

- Hypothesis 1: There is a lexical deficit associated with bilingualism.

This is the default hypothesis. Its confirmation would be consistent with the notion that bilingualism, as predicted by the amount-of-exposure accounts, inevitably leads to reduced linguistic ability in the area of the lexicon.

- Hypothesis 2: There is a lexical deficit, but it depends on language learning history.

This hypothesis posits that bilinguals who acquire both languages from birth are less likely to exhibit a lexical deficit than those who become bilingual later in life. This prediction is consistent with the timing-of-exposure frameworks.

- Hypothesis 3: There is no lexical deficit associated with bilingualism.

This hypothesis suggests that there is no systematic evidence, across studies, of a bilingual lexical deficit. To the extent that the analyzed studies include bilinguals with different learning histories, such an outcome would challenge both the amount-of-exposure and timing-of-exposure accounts.

These hypotheses speak directly to potential moderators of the lexical deficit. The first moderator is the amount of exposure that the bilingual receives in their languages. Under the assumption that input received is a major determinant of language development, this moderator should show that more exposure to a language is associated with a smaller degree of lexical deficit in that language. The second moderator is bilingualism type. Here, a distinction is made between two different types of bilinguals: (a) “simultaneous bilinguals”—that is, individuals who acquire both languages from birth—and (b) “sequential bilinguals”—that is, individuals who acquire one language from birth (i.e., the L1), and a second language (L2) after that. A third moderator, which also relates to language learning history, is age of L2 acquisition—that is, the age at which the L2 was acquired. Even though sequential bilinguals have in common that they acquired their two languages in sequence, they may differ with respect to the specific age at which the L2 was learnt. Similar to findings on other domains of language, such as syntax and phonology (e.g., Abrahamsson, 2012; Abrahamsson & Hyltenstam, 2008; Flege et al., 1999; Granena & Long, 2013; Johnson & Newport, 1989), age of L2 acquisition may be inversely correlated with nativelike lexical behaviour in the L2.

## Method

The study, including the statistical procedures, was preregistered on AsPredicted (#52642, https://aspredicted.org/VF7_8SR).

### Literature searches

**Fig. 1:**
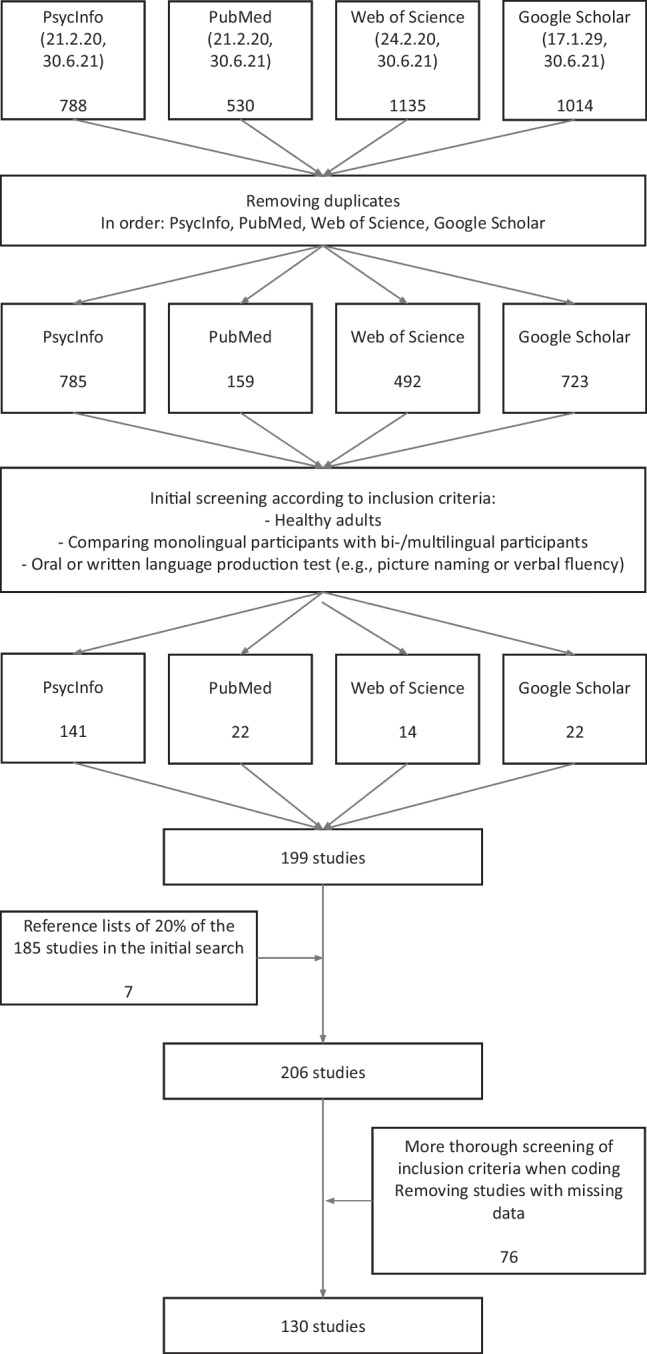
The stages of the literature search and screening process

We searched the electronic databases PsycINFO, PubMed, Web of Science, and Google Scholar by combining terms related to participant groups, such as “bilingual” and “monolingual,” and terms related to vocabulary and lexical processing and the common task paradigms to measure expressive vocabulary. Expressive vocabulary was chosen because production (as opposed to comprehension) requires higher activation thresholds of linguistic structures (e.g., Paradis, 2004). Therefore, the deficit should be most visible in production. As a number of studies that primarily focus on the bilingual cognitive advantage also report measures of expressive vocabulary from their samples, we included such terms (e.g., “executive function”) in the search strings. We first tested the sensitivity of our search strings by checking how many of 20 preidentified relevant papers would be found in the search. These papers either had the bilingual lexicon as a primary focus, or primarily concerned cognitive aspects of bilingualism and reported lexical behaviour as a bilingual background measure (for a list of papers and exact search strings, see Supplemental Table S1). Because all these papers were found, we deemed that the search strings were together sufficiently sensitive. The various stages of the search process are detailed in Fig. 1. In addition to the database searches, we screened the reference lists of 20% of the already identified and included articles for additional potentially relevant papers. The first search was conducted in February 2020, and the second search was conducted in June 2021. In case critical measures could not be obtained from a study that had been deemed relevant, the authors were contacted via email. The response rate to our emails was 59.5%, and out of these, 48.8% resulted in new data (Supplemental Table S2 lists the authors who were able to assist us with additional data).

### Inclusion and exclusion criteria

In order for a study to be included, it had to be an original study including a comparison of bilingual and monolingual participants in at least one measure of expressive vocabulary or lexical production (oral or written). We excluded studies that only reported the results of a bilingual sample because comparison with a monolingual sample is needed to measure lexical deficits related to bilingualism. We only included published peer-reviewed journal articles written in the English language.

#### Inclusion related to participants

We only included samples of adult participants (minimum mean age of the sample 18 years). We included hearing and deaf participants but excluded samples of participants with other relevant disabilities or neurocognitive impairments and diseases (e.g., dementia or aphasia), case studies, and participants undergoing brain surgery. Due to large variation in the definitions of bilingualism used in the field, we initially relied on the original study’s definitions of participants as bilingual and monolingual and coded the relevant sample characteristics (such as age of acquisition and proficiency) for more detailed moderator analyses. Samples with “novice” L2 learners were excluded based on the criterion that the bilingual participants in the original study needed to have had, on average, at least 5 years of experience of a L2 in order to be considered functionally bilingual (Johnson & Newport, 1989). Bidialectals were excluded because dialects commonly share much of the vocabulary in a language. Bimodal bilinguals were not excluded.

#### Inclusion related to tasks and measures

We included measures of lexical production, such as picture naming, verbal fluency, and synonym and antonym production. Observational and interview studies and studies only reporting physiological responses, such as brain imaging results, were excluded. As the focus of the study was on lexical production, we excluded narrative production or picture description tasks, tasks with a metalinguistic dimension (e.g., word definitions), oral repetition and reading tasks, and tasks involving morphosyntactic proficiency (e.g., cloze tests). We excluded measures from spontaneous speech and tasks that involve switching between languages. We also excluded word production tasks that are assumed to also measure other cognitive functions such as creativity and cognitive flexibility.

### Data coding

From the included studies, we coded the following variables:

- *Study characteristics*: Year of publication, country in which the study was conducted.
- *Participant characteristics*: Sample number (i.e., monolingual-bilingual group comparison, running number across original studies), mean age of each group, type of bilinguals (simultaneous, sequential), age of L2 acquisition, L1 of each group, bilinguals’ L2, bilinguals’ possible additional languages, reported exposure to the L2.^2^ Type of bilingual was determined first through how the groups were described in the paper; where relevant descriptions were lacking, groups with a mean age of acquisition above 1.5 years were coded as sequential. However, if any participants in such a group were reported as having acquired their languages from birth, the group was coded as “mixed” (because it contained both simultaneous and sequential bilinguals). When sufficient information was not provided the groups were coded as “undefined.”
- *Matching of groups*: Whether the bilingual and monolingual groups were matched for IQ and whether they were matched for education. In cases when only student populations were included, these were coded as education matched.
- *Task characteristics*: The task used, task type (letter/category fluency, picture naming), language of the task, modality of the task (oral, written).
- *Measures*: Whether the measure is based on reaction time or accuracy. *N,* mean and standard deviation for the measure in each group. Whether the same group is used in multiple comparisons.

#### Interrater reliability

The studies were coded by two raters who first coded a number of papers (e.g., 10) and then checked that their coding was uniform and resolved discrepancies through discussion. The majority of studies were then coded by a single coder. Finally, one fifth of all studies were coded by a new coder and the interrater reliability (Cohen’s kappa) was calculated. As per the pre-registration, we considered a Cohen’s kappa value of at least 0.75 as acceptable reliability. All kappa values were found to be at 0.8 or higher for key variables (see Supplemental Table S3). All discrepancies were resolved by discussion.

### Statistical analyses

We used the package metafor (Viechtbauer, 2010) for R (Version 4.0.3. for Mac; R Core Team, 2017) to conduct statistical analyses. The R script for the reported analyses, output files, and the data file can be accessed at the project’s domain at the Open Science Framework (https://osf.io/8tepa/).

We first calculated effect sizes based on the available data for bilinguals and monolinguals. Next, we evaluated the data structure and assessed the distribution of data before proceeding with synthesizing effect sizes. We further investigated how synthesized effects were affected by moderators and biases in the distribution of effect sizes.

#### Calculating effect sizes

To obtain effect sizes for the differences in monolinguals’ and bilinguals’ performances, we calculated the standardized mean difference (SMD) using the escalc function, which produces a Hedges’s *g* by adjusting the positive bias. Hedges’s *g* is a close equivalent to Cohen’s *d,* but is less biased when sample sizes are small, and is therefore often considered a corrected effect size (e.g., Borenstein et al., 2011). We set the v-type argument to “UB” for unbiased estimates of the sampling variance (Viechtbauer, 2010).

In most tasks, a higher value (e.g., score, percentage correct responses) indicated a better performance. In some tasks, a lower value (e.g., percentage incorrect, reaction times) indicated a better performance. In the latter case, we reversed effect sizes by multiplying them by −1, which allowed us to interpret all positive effect sizes as corresponding to an increased lexical deficit (i.e., monolinguals outperforming bilinguals on lexical tasks) and negative effect sizes as bilinguals outperforming monolinguals on lexical tasks.

#### Multilevel modelling

We compared the model fit indices of the one-, two-, three-, and four-level models using a likelihood-ratio test. To do this, we used the anova.rma function in metafor (Viechtbauer, 2010). Suggesting that our four-level model best fitted the data structure, all tests were statistically significant (Table 1). To evaluate the multi-level model, we used data trimmed for outliers (see Data Screening and Assessment of Bias under Results).

**Table 1:** Model fit indices, model comparison statistics, and variance components

Levels	Added levels	Model fit indices		Model comparison		Variance components		
		AIC	LogLIK	Models	LRT	σ^2^_1_	σ^2^_2_	σ^2^_3_
1. One		2343.17	−1170.58					
2. Two	Test variants	818.36	−407.28	1 vs. 2	1526.80***	0.38		
3. Three	Tests	758.36	−376.18	2 vs. 3	62.01***	0.38	0.05	
4. Four	Exp. Groups	747.29	−369.65	3 vs. 4	13.07***	0.19	0.18	0.05

AIC = Akaike information criterion; LogLik = Log-Likelihood; LRT = Likelihood-Ratio Test. The Likelihood-Ratio Test statistic is tested against a chi-square distribution with 1 degree of freedom. ***p < .001

The within-comparison dependency can be represented by an intraclass correlation coefficient (ICC), which is calculated by dividing the variance between comparisons by the sum of the variance between and within comparisons (i.e., ${\sigma}_1^2$/(${\sigma}_1^2$+${\sigma}_2^2$). If the variance within comparisons is small compared to the variance between comparisons, the ICC value is high. If, for instance, outcomes from different test variants co-vary so much that they provide the same information from the same test, the ICC will equal 1. Conversely, if they contribute completely independent information (i.e., they are no more similar than are two outcomes of two different tests), the ICC drops towards zero. In our final four-level model, the ICC for test variants within tests was .506, and the ICC for tests within comparisons of experimental groups was .789.

#### Assessment and correction of bias

We first visually inspected the data using funnel plots. In meta-analyses, funnel plots are often used to assess bias in data dispersion that might be due to publication bias or any other cause of systematic differences in outcomes between studies with low and high precision. In a typical funnel plot, effect sizes are plotted on the *x*-axis (from negative to positive, left to right) and the precision/variance on the *y*-axis (with more robust estimates higher up). Assuming that the true dispersion of effect sizes only stems from sampling bias and therefore should be symmetric, the funnel plot can be used to assess whether effect sizes are asymmetric and missing in specific areas of the plot (e.g., null effects from small studies are systematically underreported; Palmer et al., 2008). Here, we used contour-enhanced funnel plots (Peters et al., 2008), where the assessment is facilitated by shades describing statistical significance (at different levels of α) that are superimposed on the funnel plot. We plotted effect sizes against both the precision (1/*SE*) and the standard error (*SE*). Contours were added at three levels of α (*p* = .10, *p* = .05, and *p* = .005), and with a vertical reference line at Hedges’s *g* = 0.

To statistically assess and adjust for observed asymmetries in the distribution of effect sizes, we conducted a PET-PEESE analysis (Stanley & Doucouliagos, 2014). In the precision-effect test (PET) and the precision-effect test with standard error (PEESE), outcomes are regressed on their variance (*SE*^*2*^) or *SE*, respectively, in weighted least-squares regressions. In case there is a systematic bias such that studies with low precision over- or underestimate effect sizes, this bias is represented by an association between *SE* and the size and direction of the effect sizes. Modelling studies have shown that the intercept in the PEESE regression underestimates the true effect (in this case, the outcome of a hypothetical study with perfect precision). Entering the *SE*^*2*^ instead of *SE* as a predictor in a PET model provides a more accurate estimate of a true effect.

#### Moderator analyses

We analyzed the following variables as moderators of the bilingual lexical deficit: bilingualism type (i.e., simultaneous or sequential bilingualism), amount of language exposure, and age of L2 acquisition. Based on previous research on the acquisition of L2 lexis, we used age 10 as a divider between early and late acquirers (Granena & Long, 2013). By means of exploratory analyses, we further examined the moderating influence of testing language (i.e., whether bilinguals were tested in their L1 or L2), task type, and measure type (i.e., time or accuracy based).

## Results

### Descriptive results

The dataset included 478 effect sizes from 130 separate studies. Of the 478 effect sizes, 120 represented category fluency, 136 letter fluency, and 222 picture-naming tests. The bilingual groups consisted of simultaneous (5.02%), sequential (45.61%), mixed (31.38%), and undefined bilinguals (17.99%). See Supplemental Table S4 for more descriptive results.

### Data screening and assessment of bias

**Fig. 2:**
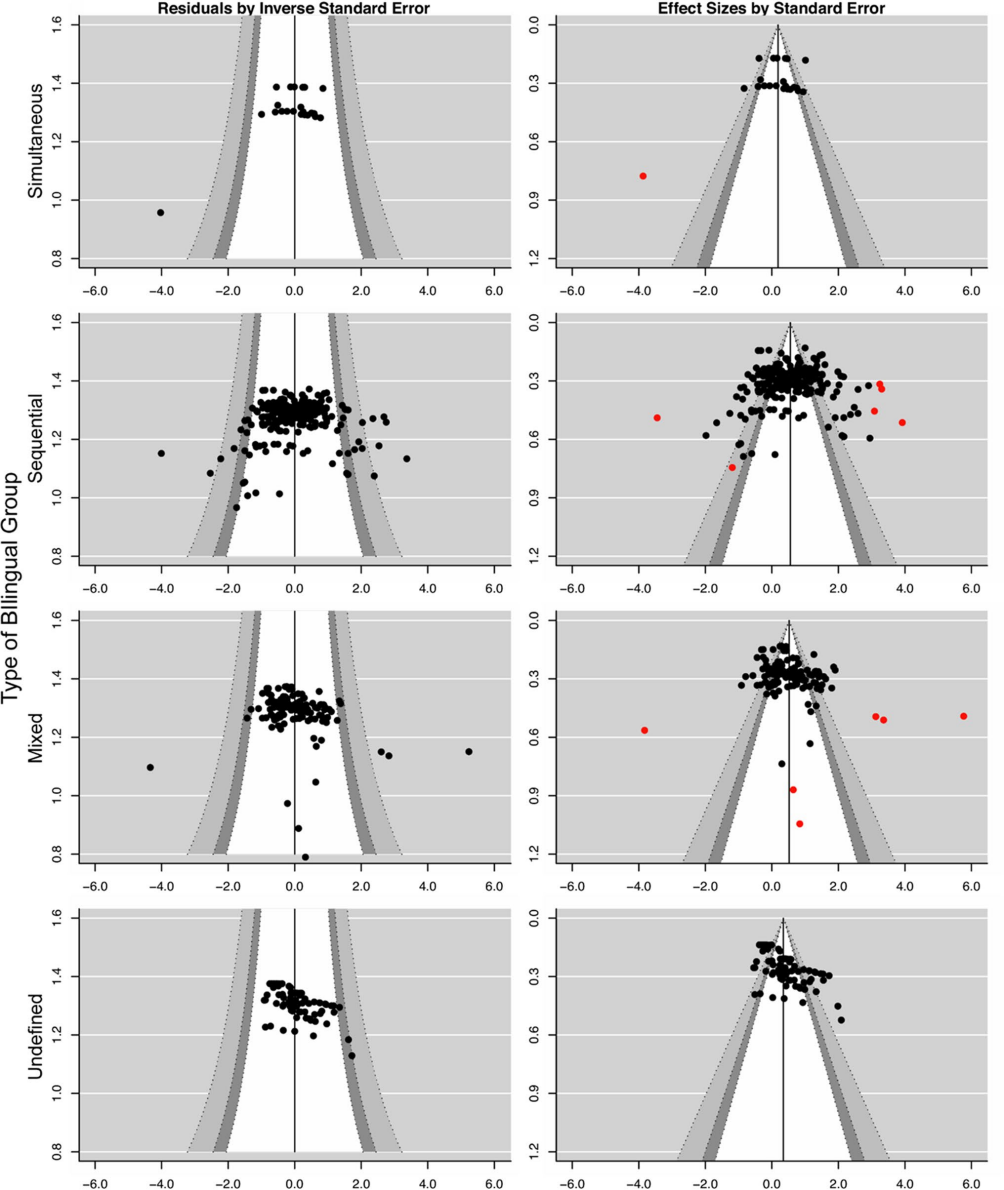
Contour-enhanced funnel plots for each type of bilingual comparison group (by row). Shades (white, light gray, dark gray) represent *p* = .10, *p* = .05., and *p* = .005, respectively. In the left column, residuals are plotted against the inverse standard error (*SE*) on the *y*-axis. The *x*-axis represents deviance from the average effect among the respective subgroup of bilingual comparison groups. In the right column, effect sizes are plotted against the standard error (*SE*). The *x*-axis represents the effect size, and effect sizes outside the range of *g* = −3.00 to *g* = 3.00 or with a variance above 0.55 are highlighted in red. Some effect sizes are outside the range of the plot

We firstly examined the distribution of effect sizes and their precision to assess asymmetries in the distribution of effect sizes such that strong positive or negative effects were overrepresented in studies with low precision, due to, for example, publication bias. To do this, we produced contour-enhanced funnel plots (Fig. 2) representing the distribution of effect sizes within each of the four types of bilingual groups. For each group, we plotted the effect sizes both against the inverse standard error (1/*SE*) and the standard error (*SE*) and on the *y*-axis. Visual inspection of these plots revealed an asymmetry in the distribution of effect sizes from comparisons including sequential and mixed bilinguals, where studies with low precision and weak or negative effects (i.e., evidence against a lexical deficit) were relatively absent. In comparisons including undefined bilinguals, there was no clear sign of asymmetry, and in comparisons including simultaneous bilinguals, one study with low precision produced a large negative effect.

The visual inspection also revealed several unrealistically large effect sizes (some of these are outside the plotted area) and studies with uncharacteristically low precision. To reduce asymmetry, increase precision in subsequent analyses, and decrease the influence of outliers on the PET-PEESE analyses, we trimmed the data by removing observations (*k* = 15; see Supplemental Table S5 for the removed effect sizes) with a variance above 0.55 and effect sizes larger than *g* = [3.00], as such observations were clearly outside the typical data distribution (which, for example, could indicate errors in the original studies).

### Lexical deficit

After trimming the data, we investigated the lexical deficit using all comparisons irrespective of the type of bilinguals used in the comparison. We found a medium effect size, *g* = 0.52 [0.42, 0.62], *p* < .001, *k* = 463, showing that monolinguals performed better than bilinguals. The test for heterogeneity was significant, *Q*[462] = 2664.83, *p* < .001. We then assessed the extent to which the observed effect was due to publication bias. Both the PET test and the PEESE test showed positive associations between the effect sizes and their *SE* and variance (*p* = .025 and *p* < .001, respectively). The adjusted effect size was smaller but remained positive and statistically significant, *g* = 0.23 [0.18, 0.27], *p* < .001.

**Fig. 3:**
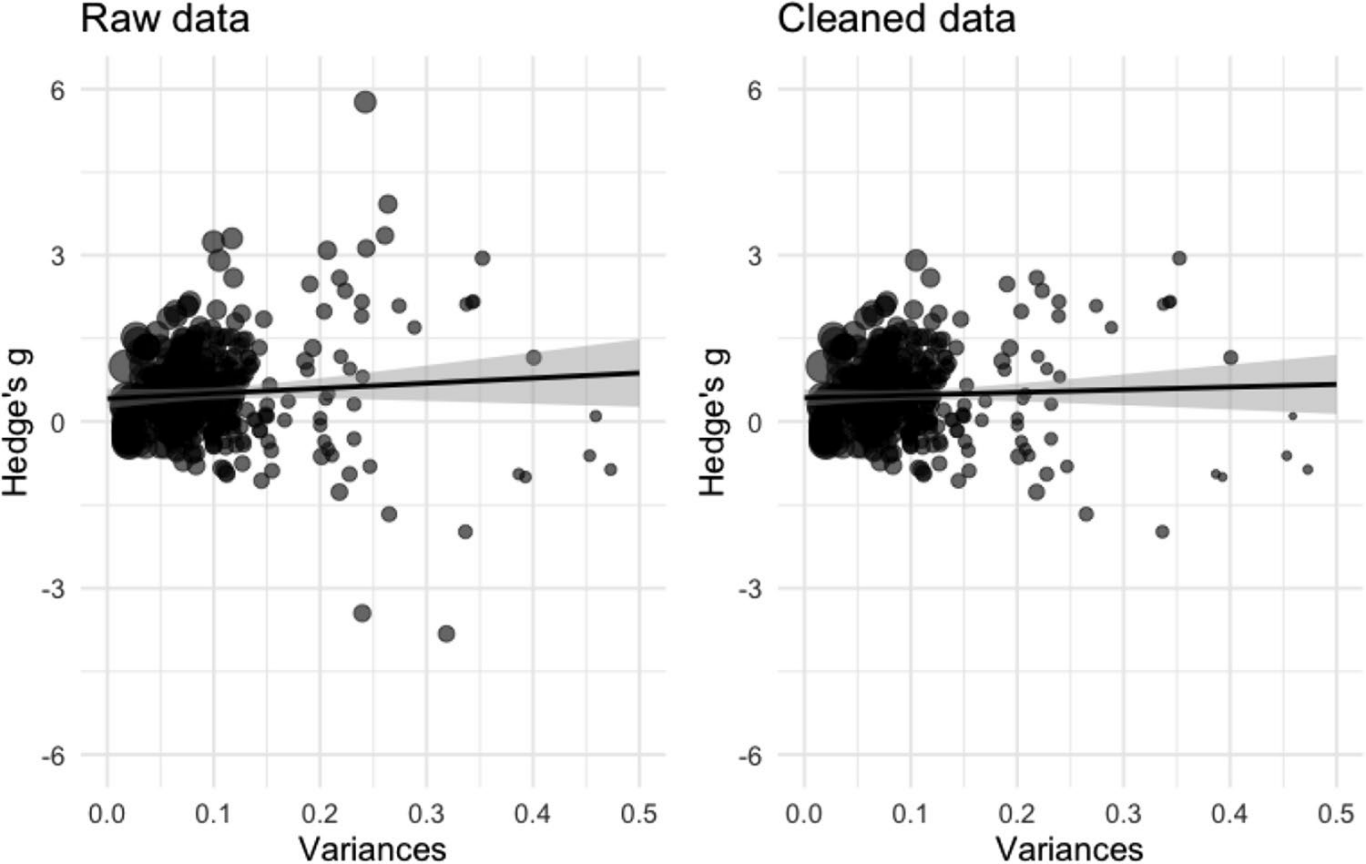
Plots of the regression slope (black line) between the effect size (*y*-axis) and the variance (*x*-axis). The shaded area gives the 95% confidence interval. The effect size of a hypothetical study with perfect precision is estimated at variance = 0. The regression for raw data is shown in the left panel; the regression for trimmed data is shown in the right panel

To follow-up on this test, we conducted a sensitivity test to investigate whether the results would be different if we used all data (i.e., also including the removed outliers). With the outliers included, both the unadjusted effect size, *g* = 0.54 [0.42, 0.66], *p* < .001, *k* = 478, and the adjusted effect size, *g* = 0.38 [0.35, 0.40], *p* < .001, were slightly larger (see Fig. 3 for plots of the association between effect sizes and their variance).

### Lexical deficit by type of bilingual group

**Fig. 4:**
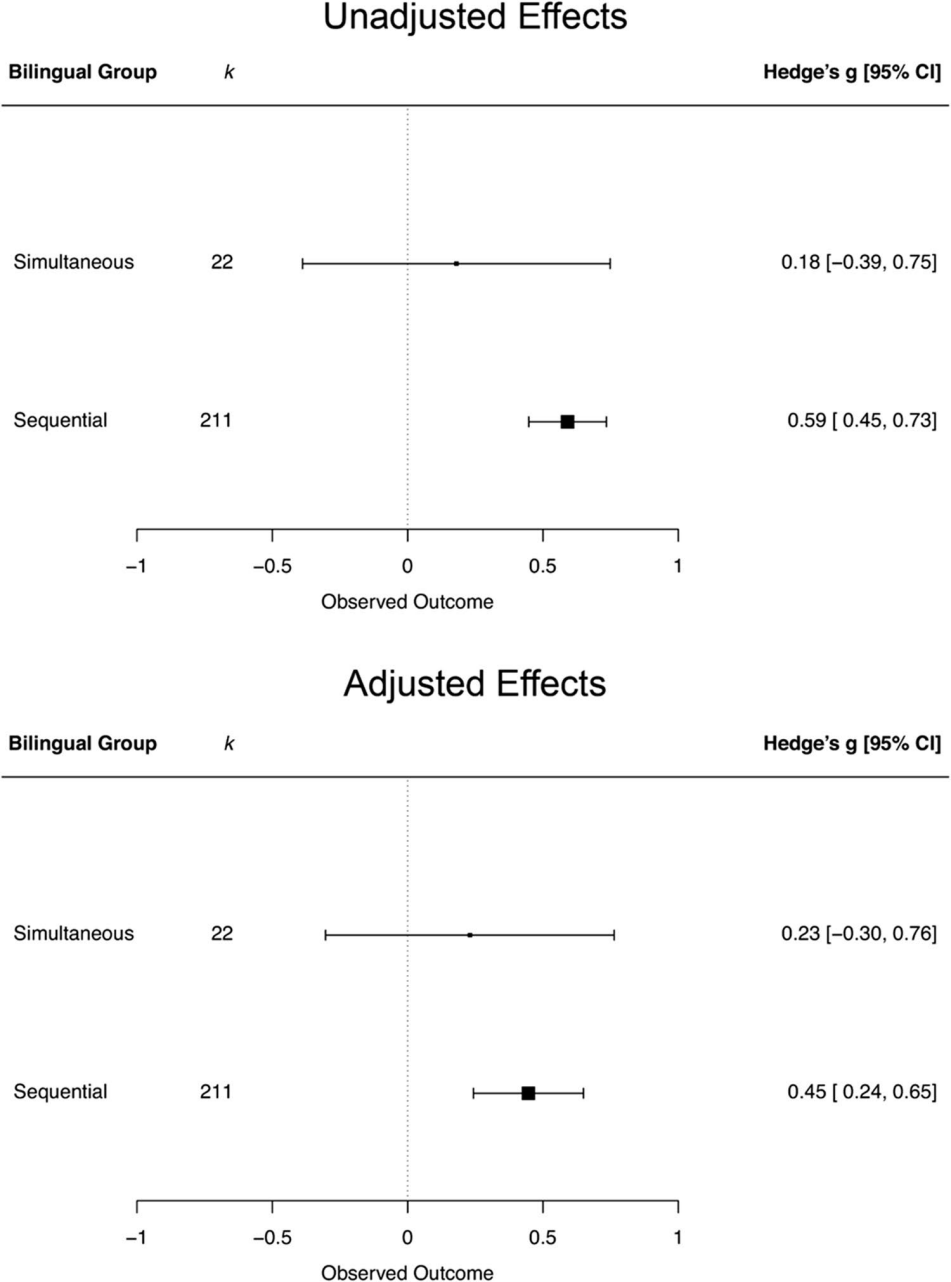
For each type of bilingual group, the figure displays synthesized effect sizes and 95% confidence intervals (CI) for the comparison between monolinguals and bilinguals. Positive values indicate monolinguals perform better; negative values indicate bilinguals perform better. *k* = number of effect sizes. The upper panel displays unadjusted effects; the bottom panel displays adjusted effects

Because there may be differences depending on the type of bilinguals that the monolinguals were compared with, we investigated whether the type of bilingual group moderated the outcome. First, we compared effect sizes for simultaneous and sequential bilinguals. We found no statistically significant difference, *Q*_*M*_ [1] = 1.89, *p* = .169, *k* = 233, and the residual heterogeneity remained significant, *Q*_*E*_ [231] = 1477.30, *p* < .001. The estimated average effect was positive for both groups. Crucially, however, the effects were statistically significant for the sequential, but not for the simultaneous bilinguals. The PET-PEESE adjusted effect sizes were smaller but remained positive (see Fig. 4).

After this, we also included the mixed and undefined bilinguals. Again, we found no statistically significant differences, *Q*_*M*_ [3] = 3.27, *p* = .352, *k* = 463. The estimates were positive also for mixed, *g* = 0.51 [0.33, 0.69], *p* < .001, *k* = 144, and undefined bilinguals, *g* = 0.37 [0.13, 0.6], *p* = .002, *k* = 86. After a PET-PEESE adjustment, estimates were slightly lower for mixed (*g* = 0.39 [0.17, 0.61], *p* < .001) and undefined bilinguals (*g* = 0.26 [−0.00, 0.53], *p* = .051).

### Testing language^3^

We then explored whether the language used to test the bilingual group (L1 or L2) moderated the effects. We found a statistically significant difference, *Q*_*M*_ [1] = 96.34, *p* < .001, *k* = 463, showing that when tests were administered in L1, the lexical deficit was smaller, *g* = 0.11 [−0.01, 022], *p* = .065, *k* = 199, than when tests were administered in L2, *g* = 0.86 [0.76, 0.97], *p* < .001, *k* = 264. The PET-PEESE adjusted values were *g* = −0.05 [−0.21, 0.12], *p* = .58 and *g* = 0.73 [0.58, 0.87], *p* < .001, respectively.

Including only sequential bilinguals in the analysis, we found an even larger difference *Q*_*M*_ = [1] = 91.69, *p* < .001, *k* = 211. When tests were administered in L1, there was no evidence of lexical deficit, *g* = 0.01 [−0.15, 0.16], *p* = .927, *k* = 104., but when tests were administered in L2, there was evidence of lexical deficit, *g* = 1.06 [0.91, 1.21], *p* < .001, *k* = 107. The pattern remained similar after adjusting for bias, *g* = −0.08 [−0.32, 0.15], *p* = .499 for L1, and *g* = 0.99 [0.79, 1.19], *p* < .001 for L2.

### Age of L2 acquisition

Next, we investigated whether there was an association between age of acquisition and lexical deficit. Because current theories and evidence on age of acquisition concern L2 development, this analysis was circumscribed to the group of sequential bilinguals tested in their L2. We did this, firstly, by categorizing age of L2 acquisition according to the reported group mean (when this information was available) into two groups: before 10 years of age (*k* = 68) and after 10 years of age (*k* = 32). We found a statistically significant difference between the groups, *Q*_*M*_ = [1] = 4.29, *p* = .038, *k* = 100. As would be expected, in the group of participants acquiring a L2 before ten years of age, *g* = 0.98 [0.78, 1.17], *p* < .001, the synthesized effect was smaller than in those who had acquired the L2 after age 10, *g* = 1.34 [1.06, 1.63], *p* < .001. After a PET-PEESE adjustment, the effect sizes were smaller but remained positive, *g* = 0.50 [0.22, 0.78], *p* = < .001 and *g* = 0.83 [0.47, 1.18], *p* < .001.

### Language exposure

We proceeded to investigate the association between language exposure and the lexical deficit. Because increased exposure to a given language is expected to lead to a lower lexical deficit in that language, but an increased deficit in the other language, we tested simultaneous and sequential bilinguals separately. The association between exposure to the testing language and lexical deficit failed to reach statistical significance in both simultaneous (*Q*_*M*_ [1] = 3.76, *p* = .052, *g* = 0.02 [−0.00, 0.05], *k* = 12) and sequential bilinguals (*Q*_*M*_ = [1] = 2.20, *p* = .138, *g* = 0.01 [−0.00, 0.01], *k* = 102). When further limiting the data to include only sequential bilinguals tested in L2, there was no association, *Q*_*M*_ [1] = 0.10, *p* = .756, *g* = −0.00 [−0.01, 0.01], *k* = 56.

### Task and measure type^4^

Finally, we explored whether measure and task type moderated the effects. Measure and task type were strongly associated, χ^2^[2] = 73.04, *p* < .001, with 31.3% of the 211 picture-naming measures being represented by reaction time, whereas only 2.5% of the 116 category-fluency measures and 2.3% of the 130 letter-fluency measures were reaction times.

We found a statistically significant difference between accuracy measures and reaction time measures, *Q*_*M*_ = [1] = 12.36, *p* < .001, *k* = 463. The synthetized effect was lower for accuracy measures, *g* = 0.49 [0.39, 0.59], *p* < .001, *k* = 392, than for reaction times, *g* = 0.74 [0.60, 0.88], *p* < .001, *k* = 72. The adjusted estimates were *g* = 0.35 [0.19, 0.52], *p* < .001 for accuracy and *g* = 0.56 [0.37, 0.75], *p* < .001 for reaction time measures.

After this, we inspected whether task type moderated the effects, and found a statistically significant difference, *Q*_*M*_ = [2] = 114.46, *p* < .001, *k* = 463. Letter fluency tasks produced the smallest effects, *g* = 0.15 [0.04, 0.27], *p* = .008, *k* = 133, followed by category fluency tasks, *g* = 0.31 [0.20, 0.43], *p* < .001, *k* = 119, and picture naming tasks, *g* = 0.75 [0.65, 0.85], *p* < .001, *k* = 211. After a PET-PEESE adjustment, the estimates were slightly lower. The estimate was not statistically significant for letter fluency, *g* = 0.04 [−0.12, 0.21], *p* = .598. For category fluency and picture naming, the estimates were *g* = 0.21 [0.05, 0.37], *p* = .012 and *g* = 0.64 [0.48, 0.79], *p* < .001, respectively.

Because measure type and task type strongly co-varied (and only picture-naming tasks included a sufficient number of both accuracy and reaction time measures), we compared accuracy and reaction time measures within picture-naming tasks. The difference between the two measure types was not statistically significant, *Q*_*M*_ [1] = 3.42, *p* = .064, *k* = 211. Reaction time measures *g* = 0.89 [0.74, 1.04], *p* < .001, *k* = 66, and accuracy measures, *g* = 0.77 [0.65, 0.89], *p* < .001, *k* = 145, produced similar effects. After adjusting for bias, the estimates were slightly smaller, *g* = 0.54 [0.32, 0.77], *p* < .001 for reaction times and *g* = 0.44 [0.24, 0.64], *p* < .001 for accuracy.

## Discussion

The present study implemented a meta-analysis to investigate whether bilingualism produces a lexical deficit. We formulated a series of alternative hypotheses that differed in their predictions as to the consistency of the lexical deficit across studies and populations. The results show that across the analyzed studies, the lexical deficit was present or more salient only under certain conditions. Crucially, these conditions were related to language learning history. Importantly, no evidence of a lexical deficit was found for simultaneous bilinguals who acquired both languages from birth. In bilinguals who acquired one language from birth and another language after that (sequential bilinguals) we found evidence of a lexical deficit under specific conditions. There was no evidence of lexical deficit in sequential bilinguals when tested in L1. A lexical deficit was present only when tested in L2. The extent of this deficit depended on age of L2 acquisition, such that the deficit was smaller in early (vs. late) learners. In addition, the lexical deficit appeared more strongly in picture-naming tasks than in fluency tasks. This result was expected because, compared to picture naming, verbal fluency (especially letter fluency) relies to a greater extent on processes other than lexical access (e.g., executive functions related to developing and maintaining retrieval strategies, see Baldo & Shimamura, 1998; Shao et al., 2014).

Overall, our findings reject the notion that bilingualism automatically brings about deviations in lexical behaviour. This interpretation is corroborated by the result that a lexical deficit was only present when sequential bilinguals were tested in their L2, not in their L1. These findings point to the possibility that the lexical deficit may be an artefact of L2 acquisition, not bilingualism. In several study designs of published research, the performance of bilingual participants tested in their L2 is compared to that of monolinguals tested in their L1. In such cases, the confounding of bilingualism versus monolingualism status with L2 versus L1 status undermines any conclusions about bilingualism being the driving factor behind verbal behaviour. Studies on the mastery of other domains of language (e.g., syntax and phonology) show that when L2 status and bilingualism are disentangled, L2 status comes out as a primary determinant of verbal behaviour (Bylund et al., 2021; Norrman & Bylund, 2016; Veríssimo et al., 2018). The current results on bilingualism type and testing language are consistent with this picture.

It would seem, then, that the findings corroborate Hypothesis 2, which predicted that language learning history would be a determinant of bilingual lexical behaviour. By extension, these results lend support to the timing-of-exposure accounts of language development, which attribute a key role to acquisition onset for the attainment of nativelike behaviour (e.g., Choi et al., 2018; Hernandez & Li, 2007; Li, 2009; Werker & Hensch, 2015). Further support for the timing-of-exposure accounts was obtained through a moderator analysis of age L2 of acquisition, which showed that individuals who started their L2 acquisition during the first decade of life were less likely to exhibit a lexical deficit than those who started past this point.

The moderator of language exposure had no measurable impact on the lexical deficit. Admittedly, it is possible that any effects of exposure were obscured by the nature of the data: because the data on this moderator were recorded at group level, the exposure values entered do not capture the actual distribution of this variable. Be that as it may, the current findings are still informative for Hypothesis 1 and, relatedly, for the amount-of-exposure accounts. The absence of a lexical deficit in simultaneous bilingualism and in L1 testing conditions shows that reduced exposure to each language, often regarded as an integral part of the bilingual experience, may not consistently incur linguistic costs.

The finding that language learning history plays an important role for lexical behaviour suggests that evidence of a lexical deficit in individuals who speak multiple languages is by and large uninformative unless learning history has been taken into account. In the absence of language learning history distinctions, it is impossible to know what underlies the observed lexical behaviour. For this reason, it is noteworthy that nearly half of the studies included in the meta-analysis either did not record learning history information or recorded the information but did not factor it into the study design. It could of course be argued that such information may have little bearing on the particular research question pursued by a given study. However, the picture that is emerging from several recent meta-analyses (besides the one at hand) is that factors of language learning history may exert a significant effect on verbal and cognitive behaviour, and neural representation (Donnelly et al., 2019; Garcia et al., 2021; Kuzmina et al., 2019; Sulpizio et al., 2020). For this reason, future studies may wish to record more details on learning history (which can easily be done with readily available background questionnaires, e.g., Anderson et al., 2017; Gullberg & Indefrey, 2003; Li et al., 2014; Marian et al., 2007) so as to anchor their findings on the characteristics of the study participants. Additionally, including this information will allow for powerful meta-analyses in the future.

### Methodological considerations

There are some methodological limitations that must be considered when interpreting the outcome of the current study. Firstly, we did not include unpublished data, which would have allowed for direct assessment of the extent of publication bias in the field. Instead, we relied on statistical assessments of bias by extrapolating the association between precision (determined by sample size and measurement error) and effect size. Studies have shown that methods to assess for publication bias, including the PET-PEESE method, are prone to errors (Carter et al., 2019), and our adjusted estimates should therefore be interpreted with some caution. Secondly, we limited the included studies to those published in English, which means that our findings are not based on a complete review of existing data. Thirdly, there was considerable heterogeneity in the distribution of included effect sizes. It is possible that the current study overlooked some important moderators.

With respect to publication bias, we found that, for studies with low precision, there was some evidence of an absence of weak or negative effect sizes that would be expected as a result of sampling error. This could mean that small studies challenging the hypothesized effect were less likely to be published than small studies supporting the hypothesized effect. The association between precision and outcome remained even after removing outliers. However, the slope was not very steep. Therefore, effect sizes largely remained positive, albeit smaller, after adjusting for the observed bias.

As a recommendation for future studies, we suggest a stronger emphasis on publishing preregistered reports, because doing so decreases publication bias against null findings. We also recommend employing more open science practices, like sharing data files and analysis scripts to facilitate reanalyses and increasing data availability for future meta-analyses (see Dal Ben et al., 2022). We also call for more stringent reporting of data, including sufficient data to calculate effect sizes and confidence intervals (i.e., sample size, means, and standard deviations for each group and outcome), as well as sufficient sample descriptives. As mentioned above, for approximately half of the included samples, we could not find sufficient information to categorize bilinguals as simultaneous or sequential.

## Conclusion

The current findings, based on a meta-analysis of 130 individual studies with 478 comparisons of monolinguals and bilinguals, show that bilingualism does not automatically incur a linguistic cost in the form of a lexical deficit. Rather, such a deficit may be a second language phenomenon. Because L2 speakers are often bilingual (i.e., to the extent that they continue using their mother tongue alongside the new language), the lexical deficit may simply *correlate* with the presence of bilingualism without there being any *causal* relationship between them. In the recent past, a case has been made for the importance of distinguishing between different types of bilingual experiences in order to understand their consequences on the human mind (e.g., Bialystok, 2017). However, until now, this argument primarily concerned the effects of bilingualism on cognitive functioning. As our study shows, it is of equal importance to take account of language learning history in order to understand the effects of bilingualism on linguistic functioning. This insight is hardly unexpected, because linguistic behaviour in the present is after all a product of language experiences in the past.

In view of both the current results and the recent findings on the cognitive advantage, we venture to say that our understanding of bilingualism is presently undergoing radical changes. The lexical deficit and the cognitive advantage might no longer be considered signatures of the bilingual experience. What is exciting about this development are the new insights it can bring regarding the potential and limits of the human capacity for language and its relationship to cognitive functioning.

## Supplementary Information

ESM 1 (PDF 34.4 kb)

ESM 2 (PDF 17.3 kb)

ESM 3 (PDF 14.4 kb)

ESM 4 (PDF 28.6 kb)

ESM 5 (PDF 36.3 kb)

## Footnotes

1 It is customary to point out that even if bilingual speakers would know fewer words in each of their languages than monolingual speakers, the total number of words they know (i.e., the vocabularies of their two languages combined) is arguably larger than that found in monolingual speakers. Moreover, the bilingual lexical deficit may be more clearly manifested in picture naming than verbal fluency tasks. This is because the latter presumably relies more on extralinguistic processes, including executive control abilities related to strategic retrieval of different words that meet certain constraints (Shao et al., 2014).

2 We included studies where exposure was reported as (or could be converted into) a percentage, but not studies reporting exposure as years or a score.

3 This moderator was not specified in the preregistration, and the analysis is thus exploratory.

4 This moderator was not specified in the preregistration and the analysis is thus exploratory.
