# Supplementary material for: Does bilingualism come with linguistic costs? A meta-analytic review of the bilingual lexical deficit
Source: Psychon Bull Rev. 2022 Nov 3;30(3):897–913. doi: 10.3758/s13423-022-02136-7 (PMC10264296; doi:10.3758/s13423-022-02136-7)
Supplement: Supplementary file 1 — (PDF 34.4 kb) [file 13423_2022_2136_MOESM1_ESM.pdf]

Table S1: Search strings used in the literature search

Database(s)	Search string
Search string PsycInfo, PubMed, Web of science[1]	*(((bilingual* or trilingual* or multilingual*) and (monolingual* or unilingual*) and (inhibition or shifting or monitoring or "working memory" or "executive function" or "executive control" or "attentional control" or "cognitive control" or vocabulary or lexic* or naming or fluency)) not children)*
Google Scholar	*bilingual monolingual "executive function" OR "executive control" OR "attentional control" OR "cognitive control" OR vocabulary OR lexical OR naming OR fluency -intitle:children -intitle:children's -intitle:review -intitle:infant -intitle:infants -intitle:toddler -intitle:machine -intitle:machines*

Notes: [1] *including filtering for peer reviewed articles and English language.*
