# Supplementary material for: Does bilingualism come with linguistic costs? A meta-analytic review of the bilingual lexical deficit
Source: Psychon Bull Rev. 2022 Nov 3;30(3):897–913. doi: 10.3758/s13423-022-02136-7 (PMC10264296; doi:10.3758/s13423-022-02136-7)
Supplement: Supplementary file 2 — (PDF 17.3 kb) [file 13423_2022_2136_MOESM2_ESM.pdf]

Table S2: Authors contributing additional data

Monika S. Schmid (University of Essex)
Kristika Kasparian and Karsten Steinhauer (McGill University)
Ping Li (Polytechnical University of Hong Kong)
F.-Xavier Alario (Aix-Marseille University)
Robert J. Hartsuiker (Ghent University)
Emilie Massa (University of Toulouse)
Tamar Degani (University of Haifa)
Hannah Claussenius-Kalman (University ofHouston)
Guosheng Ding (Beijing Normal University)
Rachel A. Ryskin (University of Illinois at Urbana–Champaign)
Arpita Bose (University of Reading)
Laiene Olaberrieta-Landa (Cruces University Hospital)
Pablo Martinez-Lage (CITA-Alzheimer Foundation)
Liubov Baladzhaeva (University of Haifa)
Longjiao Sui (Macquarie University)
María del Pilar Agustín-Llach (Universidad de la Rioja)
Anatoliy V. Kharkhurin (HSE University)
Laura-Ann Petitto (Gallaudet University)
Manon Wyn Jones and Gary M. Oppenheim (Bangor University)
Jessica K. Ljungberg (Luleå University of Technology)
Patricia M. Roberts (uOttowa)
Pui Fong Kan (University of Colorado)
Jayanthi Sasisekaran (University of Minnesota)
Eleni Peristeri (Aristotle University)
