# Supplementary material for: Does bilingualism come with linguistic costs? A meta-analytic review of the bilingual lexical deficit
Source: Psychon Bull Rev. 2022 Nov 3;30(3):897–913. doi: 10.3758/s13423-022-02136-7 (PMC10264296; doi:10.3758/s13423-022-02136-7)
Supplement: Supplementary file 3 — (PDF 14.4 kb) [file 13423_2022_2136_MOESM3_ESM.pdf]

Table S3: Cohen's Kappa values

Column	Cohen's kappa
Task type	0.98
Task domain	0.94
Test language	0.92
Data type	0.90
Monolingual N	0.96
Monolingual mean	0.80
Monolingual sd	0.88
Bilingual N	0.90
Bilingual mean	0.80
Bilingual SD	0.93
