# Supplementary material for: Does bilingualism come with linguistic costs? A meta-analytic review of the bilingual lexical deficit
Source: Psychon Bull Rev. 2022 Nov 3;30(3):897–913. doi: 10.3758/s13423-022-02136-7 (PMC10264296; doi:10.3758/s13423-022-02136-7)
Supplement: Supplementary file 4 — (PDF 28.6 kb) [file 13423_2022_2136_MOESM4_ESM.pdf]

Table S4: Participant-Related Information of the Included Studies

Study	Sample (# ES)	Bilingual group	Sample description	Country	Age group	L1/L2	Age	AoA
Abutalebi et al. (2012)	1 (1)	Mixed	Healthy adults	Italy	Younger	German/Italian	23.35	
Abutalebi et al. (2013)	1 (1)	Mixed	University students	Italy	Younger	German/Italian		
	1 (1)	Sequential	Adults	Italy	Younger	German/Italian	23.35	6.00
Akhavan et al. (2020)	1 (1)	Mixed	University students	USA	Younger	Spanish/English	21.60	2.10
Anderson et al. (2017)	1 (3)	Undefined	Healthy older adults	Canada	Older	Mixed/English	74.70	
Anderson et al. (2018)	1 (1)	Mixed	Young adults	Canada	Younger	English/French	22.60	4.47
Ansaldo et al. (2015)	1 (2)	Sequential	Older adults	Canada	Older	French/Mixed	74.20	17.25
Ardila et al. (2019)	1 (2)	Sequential	College students	USA	Younger	Spanish/English	23.60	
	2 (2)	Mixed	College students	USA	Younger	Spanish/English	23.70	
Baladzhaeva and Laufer (2018)	1 (2)	Sequential	Control group	Israel/Russia/Kazakhstan	Older	Russian/Hebrew	71.48	53.90
	2 (2)	Sequential	Hebrew- group	Israel	Older	Russian/Hebrew	71.48	53.90
Barbu et al. (2020)	1 (1)	Undefined	Adults	Belgium	Younger	German/French	25.73	
	2 (1)	Undefined	Adults	Belgium	Younger	German/French	24.90	
Baus et al. (2020)	1 (2)	Sequential	University students	Spain	Younger	Spanish/Basque	22.00	3.00
	2 (2)	Sequential	University students	Spain	Younger	Basque/Spanish	24.00	5.00
Bellegarda and Macizo (2021)	1 (1)	Sequential	University student	Spain	Younger	Mixed/Spanish	26.58	13.96
Bennett and Verney (2019)	1 (1)	Undefined	University students	USA	Younger	Spanish/English	20.00	
Berroir et al. (2017)	1 (3)	Sequential	Elderly adults	Canada	Older	French/English	74.20	16.70
Bialystok et al. (2008)	1 (4)	Mixed	Younger adults	Canada	Younger	Mixed/English	19.70	
	2 (4)	Mixed	Older adults	Canada	Older	Mixed/English	68.30	
	1 (3)	Mixed	University students (Study 1)	Canada	Younger	Mixed/English	19.70	4.00
	2 (3)	Mixed	Young adults (Study 2)	Canada	Younger	Mixed/English	21.40	
Bice and Kroll (2021)	1 (1)	Mixed	University students	USA	Younger	Spanish/English	19.60	3.54
Blumenfeld and Marian (2014)	1 (2)	Sequential	Young adults (Study 1)	USA	Younger	English/Spanish	22.00	2.90
	2 (2)	Sequential	Young adults (Study 2)	USA	Younger	Spanish/English	21.70	4.40
Blumenfeld et al. (2016)	1 (2)	Sequential	University students	USA	Younger	English/Spanish	21.96	1.96
Bogulski et al. (2015)	1 (2)	Sequential	University students	Canada	Younger	English/French	21.20	
Bogulski et al. (2019)	1 (1)	Sequential	Adults	USA	Younger	English/Spanish	23.23	11.05
	2 (1)	Mixed	Adults	USA	Younger	Spanish/English	26.68	6.58
	3 (1)	Sequential	Adults	USA	Younger	Mandarin Chinese/English	24.53	11.71
Borodkin et al. (2016)	1 (1)	Sequential	Undergraduate students	Israel	Younger	English/Hebrew	22.63	7.32
Botezatu et al. (2021)	1 (1)	Sequential	University students	USA	Younger	Mixed/English	22.30	
Bradley et al. (2013)	1 (1)	Sequential	Adults	USA	Younger	Spanish/English	21.75	4.55
Broos et al. (2019)	1 (1)	Sequential	University students	Belgium/UK	Younger	Dutch/English	23.00	
Broos et al. (2021)	1 (1)	Sequential	University students	UK/Netherlands	Younger	Dutch/English	19.00	10.80
Clare et al. (2016)	1 (1)	Mixed	Older adults	UK	Older	Welsh/English	74.32	
Claussenius-Kalman et al. (2020)	1 (1)	Sequential	Healthy adults	USA	Younger	Spanish/English	22.09	5.19
	2 (1)	Sequential	Healthy adults	USA	Younger	Spanish/English	25.75	13.84
Costa et al. (2000)	1 (2)	Sequential	University students	Spain	Younger	Catalan/Spanish		
Costa et al. (2003)	1 (1)	Sequential	Young adults	Italy	Younger	Croatian/Italian	26.00	5.00
	2 (1)	Sequential	Young adults	Italy	Younger	Croatian/Italian	23.00	8.00
	3 (1)	Sequential	Young adults	Spain	Younger	Spanish/Catalan	21.00	5.00
	4 (1)	Sequential	Young adults	Spain	Younger	Catalan/Spanish	21.00	5.00
	5 (1)	Sequential	Young adults	France	Younger	Italian/French	26.00	6.00
de Bruin et al. (2016)	1 (2)	Sequential	Older adults	UK	Older	Gaelic/English	71.86	4.30
	2 (2)	Undefined	Older adults	UK	Older	Gaelic/English	70.50	3.80
Degani and Tokowicz (2013)	1 (1)	Sequential	Adults	USA	Younger	Spanish/English	33.63	10.98

Study	Sample (# ES)	Bilingual group	Sample description	Country	Age group	L1/L2	Age	AoA
	2 (1)	Sequential	Adults	USA	Younger	English/Spanish	23.83	12.10
Egan et al. (2019)	1 (1)	Mixed	University students	Wales, UK	Younger	Welsh/English	22.66	2.00
Emmorey et al. (2013)	1 (1)	Simultaneous	Adults	USA	Younger	English/American Sign Language	27.00	0.00
	2 (1)	Sequential	Adults	USA	Younger	English/American Sign Language	36.00	16.40
	3 (1)	Simultaneous	Adults	USA	Younger	English/American Sign Language	27.00	0.00
	4 (1)	Sequential	Adults	USA	Younger	English/American Sign Language	36.00	16.40
Estanga et al. (2017)	1 (3)	Sequential	Healthy adults	Spain	Younger	Spanish/Euskera	57.56	9.70
	2 (3)	Mixed	Healthy adults	Spain	Younger	Spanish/Euskera	56.82	2.96
Fernandez et al. (2013)	1 (1)	Sequential	Adults	USA	Younger	Spanish/English	20.46	6.00
Filippi et al. (2022)	1 (2)	Mixed	Healthy adults	UK	Younger	Mixed/Mixed	37.80	
Francis et al. (2018)	1 (1)	Mixed	Adults	USA	Younger	Spanish/English	22.00	4.90
	2 (1)	Sequential	Adults	USA	Younger	Spanish/English	21.00	7.20
Friesen et al. (2015)	1 (2)	Mixed	Younger adults	Canada	Younger	English/Mixed	21.10	2.90
	2 (2)	Sequential	Older adults	Canada	Older	English/Mixed	71.10	8.80
Giezen and Emmorey (2017)	1 (1)	Simultaneous	Adults	USA	Younger	English/American Sign Language	25.60	
Gollan and Brown (2006)	1 (1)	Sequential	University students	USA	Younger	Spanish/English	20.44	4.14
Gollan et al. (2002)	1 (3)	Mixed	Adults	USA?	Younger	Spanish/English	20.00	3.40
Gollan et al. (2005)	1 (1)	Mixed	University students (Study 1)	USA	Younger	Spanish/English	20.30	4.00
	2 (1)	Mixed	University students (Study 2)	USA	Younger	Spanish/English	20.20	3.50
	1 (1)	Sequential	University students	USA	Younger	Spanish/English	21.20	3.60
Gollan et al. (2008)	1 (1)	Mixed	Undergraduate students	USA	Younger	Spanish/English	19.30	2.70
	2 (1)	Mixed	Older adults	USA	Older	Spanish/English	74.90	4.60
Gollan et al. (2011)	1 (1)	Sequential	University students	USA/Belgium	Younger	Spanish/English	20.00	3.70
	2 (1)	Sequential	University students	USA/Belgium	Younger	Dutch/English	19.30	10.80
Gollan et al. (2012)	1 (1)	Mixed	Undergraduate students	USA	Younger	English/Spanish	20.70	3.00
	2 (1)	Mixed	Undergraduate students	USA	Younger	English/Spanish	23.10	2.90
	3 (1)	Mixed	Undergraduate students	USA	Younger	Spanish/English	19.30	6.40
Hirsh et al. (2003)	1 (1)	Sequential	Adults	UK	Younger	Spanish/English	22.10	11.00
Ivanova et al. (2013)	1 (2)	Sequential	Healthy adults	USA	Older	Spanish/English	80.20	9.40
Johns et al. (2016)	1 (3)	Undefined	Younger adults	Canada	Younger	English/French	21.68	
	2 (3)	Undefined	Adults	Canada	Older	English/French	70.62	
Kalia et al. (2014)	1 (1)	Mixed	Undergraduate students	USA	Younger	Mixed/English	19.82	
	2 (1)	Sequential	Undergraduate students	USA	Younger	Mixed/English	19.72	
Kan and Sadagopan (2014)	1 (1)	Sequential	Young adults	USA	Younger	English/Spanish	22.40	6.27
Kasparian and Steinhauer (2016)	1 (1)	Sequential	Adults	Canada/Italy	Younger	Italian/English	36.00	28.20
Kasparian and Steinhauer (2017)	1 (1)	Sequential	Adults	Canada/Italy	Younger	Italian/English	36.00	28.20
Kaushanskaya and Marian (2009)	1 (1)	Sequential	University students	USA	Younger	English/Spanish	20.83	5.44
	2 (1)	Sequential	University students	USA	Younger	English/Mandarin Chinese	21.10	2.21
	1 (1)	Mixed	University students	USA	Younger	English/Spanish	20.83	5.44
Kaushanskaya et al. (2011)	1 (1)	Sequential	Adults	USA	Younger	English/Spanish	22.12	7.39
Kharkhurin (2008)	1 (1)	Sequential	College students	USA	Younger	Russian/English	21.57	9.27
Kharkhurin (2009)	1 (1)	Sequential	University students	United Arab Emirates	Younger	Farsi/English	22.38	8.47
Kharkhurin (2010)	1 (1)	Sequential	Young adults	USA	Younger	Russian/English	21.57	9.27
	2 (1)	Sequential	Young adults	Iran	Younger	Farsi/English	22.38	8.47
	1 (1)	Sequential	College students	USA	Younger	Russian/English	21.57	9.27
Kharkhurin (2017)	1 (1)	Sequential	University students	Russia	Younger	Russian/English	19.69	9.46
Kousaie et al. (2014)	1 (3)	Mixed	Younger adults	Canada	Younger	English/French	21.49	
	2 (3)	Mixed	Younger adults	Canada	Younger	English/French	21.49	
	3 (3)	Mixed	Older adults	Canada	Older	French/English	70.69	

Study	Sample (# ES)	Bilingual group	Sample description	Country	Age group	L1/L2	Age	AoA
	4 (3)	Mixed	Older adults	Canada	Older	French/English	70.69	
Kovelman et al. (2009)	1 (1)	Simultaneous	Adults	USA	Younger	American Sign Language/English	24.00	0.00
	2 (1)	Simultaneous	Adults	USA	Younger	American Sign Language/English	24.00	0.00
Kreiner and Degani (2015)	1 (1)	Sequential	Adults	Israel	Younger	Russian/Hebrew	26.88	12.92
	2 (1)	Mixed	Adults	Israel	Younger	Russian/Hebrew	24.46	3.19
Li et al. (2013)	1 (1)	Sequential	University students	USA	Younger	Mandarin Chinese/English	24.44	10.64
Li et al. (2015)	1 (1)	Sequential	Healthy adults	China	Younger	Mandarin Chinese/Chinese Sign Language	49.00	19.07
Li et al. (2017)	1 (1)	Mixed	Undergraduate students	USA	Younger	Spanish/English	20.40	3.80
	2 (1)	Mixed	Undergraduate students	USA	Younger	Mandarin/English	20.10	4.00
Lin and Lin (2016)	1 (1)	Sequential	Adults	USA	Younger	Mandarin Chinese/English	35.50	11.70
	2 (1)	Sequential	Adults	USA	Younger	Spanish/English	20.70	2.80
Ljungberg et al. (2013)	1 (2)	Sequential	Adults	Sweden	Younger	Swedish/English	35.00	
	2 (2)	Sequential	Adults	Sweden	Younger	Swedish/English	40.00	
	3 (2)	Sequential	Adults	Sweden	Younger	Swedish/English	45.00	
	4 (2)	Sequential	Adults	Sweden	Younger	Swedish/English	50.00	
	5 (2)	Sequential	Adults	Sweden	Younger	Swedish/English	55.00	
	6 (2)	Sequential	Adults	Sweden	Younger	Swedish/English	60.00	
	7 (2)	Sequential	Adults	Sweden	Younger	Swedish/English	65.00	
	8 (2)	Sequential	Adults	Sweden	Younger	Swedish/English	70.00	
Ljungberg et al. (2020)	1 (2)	Mixed	Adults	Sweden	Younger	Swedish/Finnish	57.50	9.00
	2 (2)	Mixed	Adults	Sweden	Younger	Swedish/English	57.50	9.00
Luo et al. (2010)	1 (3)	Sequential	Adults	Canada	Younger	English/Mixed	21.10	2.90
	2 (3)	Sequential	Adults	Canada	Younger	English/Mixed	20.30	4.30
Massa et al. (2020)	1 (2)	Sequential	Young adults	France	Younger	French/Italian	25.60	11.10
	2 (2)	Sequential	Older adults	France	Older	French/Italian	72.30	10.20
Milman et al. (2018)	1 (1)	Sequential	Community-dwelling adults	USA	Younger	Spanish/English	49.00	15.20
	2 (1)	Sequential	Community-dwelling adults	USA	Younger	Asian Indian languages/English	58.40	5.80
Misdraji-Hammond et al. (2015)	1 (1)	Mixed	Adults	USA	Younger	Spanish/English	29.27	
Mizrahi et al. (2021)	1 (1)	Undefined	Adults	USA	Younger	Spanish/English	19.80	3.20
	2 (1)	Undefined	Adults	USA	Younger	Spanish/English	20.20	2.80
	3 (1)	Undefined	Adults	USA	Younger	Spanish/English	20.10	2.60
	4 (1)	Undefined	Adults	USA	Younger	Spanish/English	20.10	3.40
	5 (1)	Undefined	Adults	USA	Younger	Spanish/English	19.80	3.20
	6 (1)	Undefined	Adults	USA	Younger	Spanish/English	20.20	2.80
	7 (1)	Undefined	Adults	USA	Younger	Spanish/English	20.10	2.60
	8 (1)	Undefined	Adults	USA	Younger	Spanish/English	20.10	3.40
Mor et al. (2015)	1 (2)	Mixed	University students	Israel	Younger	Russian/Hebrew	24.80	
Morrison and Taler (2020)	1 (3)	Undefined	University students	Canada	Younger	English/French	20.54	4.57
	2 (3)	Undefined	Older adults	Canada	Older	English/French	72.27	6.75
Navarro-Torres et al. (2019)	1 (1)	Sequential	University students	USA	Younger	Mixed/English	23.96	6.29
Olabarrieta-Landa Laiene et al. (2019)	1 (1)	Undefined	Healthy adults	Spain	Younger	Basque/Spanish	45.57	4.05
	2 (1)	Undefined	Healthy adults	Spain	Younger	Catalan/Spanish	49.45	4.02
Olsen et al. (2015)	1 (2)	Undefined	Older adults	Canada	Older	English/Mixed	69.90	
Oschwald et al. (2018)	1 (1)	Undefined	Young adults	Switzerland	Younger	German/Indo-European language family	22.50	
	2 (1)	Undefined	Young adults	Switzerland	Younger	German/Non-Indo-European language family	23.38	
Paap et al. (2017)	1 (4)	Mixed	Undergraduate students	USA	Younger	English/Mixed	21.14	
Palomar-García et al. (2015)	1 (1)	Mixed	Undergraduate students	Spain	Younger	Spanish/Catalan	20.22	2.70
Paplikar et al. (2021)	1 (1)	Undefined	Adults	India	Younger	Hindi/Mixed	50.80	
	2 (1)	Undefined	Adults	India	Younger	Hindi/Mixed	50.80	

Study	Sample (# ES)	Bilingual group	Sample description	Country	Age group	L1/L2	Age	AoA
	3 (1)	Undefined	Adults	India	Younger	Bengali/Mixed	53.70	
	4 (1)	Undefined	Adults	India	Younger	Bengali/Mixed	53.70	
	5 (1)	Undefined	Adults	India	Younger	Telugu/Mixed	55.00	
	6 (1)	Undefined	Adults	India	Younger	Telugu/Mixed	55.00	
	7 (1)	Undefined	Adults	India	Younger	Kannada/Mixed	61.90	
	8 (1)	Undefined	Adults	India	Younger	Kannada/Mixed	61.90	
	9 (1)	Undefined	Adults	India	Younger	Malayalam/Mixed	57.10	
	10 (1)	Undefined	Adults	India	Younger	Malayalam/Mixed	57.10	
Patra et al. (2020)	1 (2)	Sequential	Young adults	UK	Younger	Bengali/English	32.80	7.18
Pelham and Abrams (2013)	1 (1)	Sequential	University students	USA	Younger	Spanish/English	22.00	11.60
	2 (1)	Mixed	University students	USA	Younger	Spanish/English	20.30	3.30
Peñaloza et al. (2019)	1 (1)	Mixed	Healthy adults	USA?	Younger	Catalan/Spanish	30.50	6.30
	2 (1)	Mixed	Healthy adults	USA?	Younger	Spanish/English	44.28	13.24
	3 (1)	Mixed	Healthy adults	USA?	Younger	English/Spanish	30.50	6.30
	4 (1)	Mixed	Healthy adults	USA?	Younger	Spanish/English	44.28	13.24
Peristeri et al. (2018)	1 (1)	Sequential	Adults	Greece	Younger	Serbian/Greek	29.60	17.30
	2 (1)	Mixed	Adults	Greece	Younger	Serbian/Greek	24.50	1.30
Portocarrero et al. (2007)	1 (3)	Sequential	Undergraduate college students	USA	Younger	Mixed/English	19.00	9.50
Prior and Gollan (2011)	1 (1)	Sequential	Undergraduate students	USA	Younger	Spanish/English	20.00	2.70
	2 (1)	Sequential	Undergraduate students	USA	Younger	Mandarin Chinese/English	19.40	2.70
Pyers et al. (2009)	1 (1)	Undefined	Adults	USA	Younger	American Sign Language/English	23.41	0.13
	2 (1)	Undefined	Adults	USA	Younger	Spanish/English	20.64	0.27
Roberts et al. (2002)	1 (1)	Mixed	Young adults	Canada/USA	Younger	Spanish/English	39.60	5.50
	2 (1)	Mixed	Young adults	Canada/USA	Younger	French/English	34.90	5.50
Rodriguez-Fornells et al. (2005)	1 (1)	Mixed	University students	Germany	Younger	Spanish/German	26.10	
Rosselli et al. (2000)	1 (3)	Mixed	Older adults	USA	Older	Spanish/English	60.60	18.85
	2 (3)	Mixed	Older adults	USA	Older	Spanish/English	60.60	18.85
Rosselli et al. (2002)	1 (2)	Mixed	Adults	USA	Older	Spanish/English		18.85
	2 (2)	Mixed	Adults	USA	Older	Spanish/English		18.85
Rosselli et al. (2016)	1 (1)	Sequential	Undergraduate students	USA	Younger	English/Spanish	26.80	6.60
	2 (1)	Sequential	Undergraduate students	USA	Younger	English/Spanish	25.20	8.20
	3 (1)	Sequential	Undergraduate students	USA	Younger	English/Spanish	26.90	10.40
	4 (1)	Sequential	Undergraduate students	USA	Younger	English/Spanish	26.80	6.60
	5 (1)	Sequential	Undergraduate students	USA	Younger	English/Spanish	25.20	8.20
	6 (1)	Sequential	Undergraduate students	USA	Younger	English/Spanish	26.90	10.40
Runnqvist et al. (2013)	1 (1)	Sequential	Adults	USA?	Younger	Mandarin Chinese/English	20.21	3.13
	2 (1)	Sequential	Adults	USA?	Younger	Spanish/English	21.44	2.95
Ryskin et al. (2014)	1 (1)	Undefined	University students	USA	Younger	English/Mixed	19.80	0.74
Sadat et al. (2012)	1 (1)	Sequential	Undergraduate students	Spain	Younger	Spanish/Catalan	21.10	3.43
	2 (1)	Sequential	Undergraduate students	Spain	Younger	Spanish/Catalan	21.00	4.80
Sadat et al. (2016)	1 (1)	Mixed	Undergraduate students	Spain	Younger	Spanish/Catalan	21.00	1.00
	2 (1)	Mixed	Undergraduate students	Spain	Younger	Catalan/Spanish	22.00	1.00
Salvatierra and Rosselli (2010)	1 (1)	Sequential	College students	USA	Younger	Spanish/English	26.67	11.00
	2 (1)	Sequential	Students' family members and friends	USA	Older	Spanish/English	64.84	19.74
Sandoval et al. (2010)	1 (2)	Mixed	University students	USA	Younger	Spanish/English	20.33	2.09
Sasisekaran and Weisberg (2013)	1 (1)	Sequential	Undergraduate students	USA	Younger	English/Mixed	22.70	14.00
Savoie et al. (2019)	1 (1)	Undefined	Community-dwelling individuals	Canada	Younger	French/English	43.81	
Schmid (2014)	1 (1)	Sequential	Adults	Germany/Canada	Older	German/English	63.20	26.20
	2 (1)	Sequential	Adults	Germany	Older	English/German	51.90	25.30

Study	Sample (# ES)	Bilingual group	Sample description	Country	Age group	L1/L2	Age	AoA
Schmidtke (2014)	1 (1)	Mixed	Young adults	USA	Younger	Spanish/English	21.60	3.50
	2 (1)	Sequential	Young adults	USA	Younger	Spanish/English	24.10	6.70
Schmidtke (2016)	1 (1)	Mixed	Adults	USA	Younger	Spanish/English	20.80	4.40
Seçer (2016)	1 (1)	Sequential	Younger adults	Cyprus	Younger	Turkish/English	22.39	11.83
Segal and Gollan (2018)	1 (1)	Sequential	Undergraduate students	USA	Younger	Spanish/English	19.80	3.80
Sehyr et al. (2018)	1 (1)	Sequential	Young adults	USA	Younger	English/American Sign Language	29.50	15.00
	2 (1)	Sequential	Young adults	USA	Younger	English/American Sign Language	29.50	15.00
	3 (1)	Simultaneous	Young adults	USA	Younger	English/American Sign Language	29.00	
	4 (1)	Simultaneous	Young adults	USA	Younger	English/American Sign Language	29.00	
Sheppard et al. (2016)	1 (3)	Sequential	Undergraduate students	Canada	Younger	English/French	21.57	4.00
	2 (3)	Sequential	Undergraduate students	Canada	Younger	English/French	21.57	4.00
	3 (3)	Sequential	Older adults	Canada	Older	French/English	70.69	6.00
	4 (3)	Sequential	Older adults	Canada	Older	French/English	70.69	6.00
Soltani et al. (2019)	1 (2)	Undefined	Older adults	Iran	Older	Arabic/Persian	72.80	
Stasenko and Gollan (2019)	1 (1)	Sequential	University students	USA	Younger	Spanish/English	20.30	3.50
Stasenko et al. (2017)	1 (1)	Mixed	Undergraduate students	USA	Younger	Spanish/English	20.30	3.30
Sullivan et al. (2018)	1 (1)	Sequential	Undergraduate students	Canada	Younger	Mixed/English	20.70	61.10
	2 (1)	Sequential	Undergraduate students	Canada	Younger	Mixed/English	21.20	5.80
	3 (1)	Sequential	Community members	Canada	Older	Mixed/English	74.10	5.50
	4 (1)	Sequential	Community members	Canada	Older	Mixed/English	71.60	8.30
Sundaray et al. (2018)	1 (2)	Mixed	Undergraduate students	UK	Younger	Tamil/English	20.93	
	2 (2)	Sequential	Older adults	UK	Older	Tamil/English	67.01	
Taler et al. (2013)	1 (3)	Undefined	Adults	Canada	Younger	English/French	21.53	
Taler et al. (2016)	1 (3)	Undefined	Undergraduate students	Canada	Younger	English/French	21.11	
Tao et al. (2015)	1 (2)	Simultaneous	Undergraduate students	USA	Younger	English/Spanish	20.90	
	2 (3)	Simultaneous	Undergraduate students	USA	Younger	English/Mandarin Chinese	19.80	
Vega-Mendoza et al. (2015)	1 (2)	Sequential	University students (Experiment 1)	UK	Younger	English/Spanish	22.44	
	2 (2)	Sequential	University students (Experiment 1)	UK	Younger	English/Spanish	20.82	
Woumans et al. (2015)	1 (1)	Mixed	University students	Belgium	Younger	Dutch/French	21.10	2.60
	2 (1)	Sequential	University students	Belgium	Younger	Dutch/French	22.50	8.60
Woumans et al. (2019)	1 (1)	Mixed	Undergraduate students	Belgium	Younger	Dutch/Mixed	19.82	4.72
Yoo and Kaushanskaya (2012)	1 (1)	Sequential	Young adults	USA?	Younger	Korean/English	28.43	10.38
Zirnstein et al. (2018)	1 (1)	Undefined	Young adults	USA	Younger	Mandarin Chinese/English	23.00	
Zirnstein et al. (2019)	1 (1)	Mixed	Older adults	Canada	Older	English/Mixed		
Zou et al. (2012)	1 (1)	Sequential	Healthy adults, experiment 1	China	Younger	Mandarin Chinese/Chinese Sign Language	49.00	19.00
	1 (1)	Sequential	Adults	China	Younger	Mandarin Chinese/Chinese Sign Language	49.00	19.00
	1 (1)	Sequential	Adults	Sweden	Younger	Spanish/Swedish	32.20	5.15
	1 (1)	Mixed	Undergraduate students	USA	Younger	Mixed/English		5.80
