# Supplementary material for: Does bilingualism come with linguistic costs? A meta-analytic review of the bilingual lexical deficit
Source: Psychon Bull Rev. 2022 Nov 3;30(3):897–913. doi: 10.3758/s13423-022-02136-7 (PMC10264296; doi:10.3758/s13423-022-02136-7)
Supplement: Supplementary file 5 — (PDF 36.3 kb) [file 13423_2022_2136_MOESM5_ESM.pdf]

Study	Sample	N (m/b)	Domain	Task	Data type	M	SD	M	SD	Hedges' g	Notes
Abutalebi et al. (2012)	1	14/17	Oral	Picture naming	Percent incorrect	2.46	1.13	2.76	1.60	0.21 (0.13)	R
Abutalebi et al. (2013)	1	14/14	Oral	Picture naming	Percent incorrect	5.21	1.38	4.86	2.62	-0.16 (0.14)	R
Abutalebi et al. (2013)	1	14/14	Oral	Picture naming	Percent incorrect	5.21	1.38	4.86	2.62	-0.16 (0.14)	R
Akhavan et al. (2020)	1	19/21	Oral	Category fluency	Score	54.70	11.00	52.70	9.60	0.19 (0.10)	
Anderson et al. (2017)	1	18/16	Oral	Letter fluency	Score	13.90	3.10	12.40	3.80	0.43 (0.12)	
		18/16	Oral	Category fluency	Score	13.20	3.60	9.80	4.20	0.85 (0.13)	
		18/16	Oral	Category fluency	Score	13.60	2.90	10.10	4.00	0.99 (0.13)	
Anderson et al. (2018)	1	14/15	Oral	Picture naming	Percent correct	95.10	5.70	94.90	5.10	0.04 (0.14)	
Ansaldo et al. (2015)	1	10/10	Oral	Category fluency	Score	43.70	7.50	49.30	10.10	-0.60 (0.21)	
		10/10	Oral	Picture naming	Percent correct	94.50	37.60	92.40	30.90	0.06 (0.20)	
Ardila et al. (2019)	1	20/24	Oral	Category fluency	Score	34.75	5.97	32.50	6.30	0.36 (0.09)	
		20/24	Oral	Letter fluency	Score	11.21	1.45	39.50	10.80	-3.45 (0.24)	O
	2	20/18	Oral	Category fluency	Score	34.75	5.97	34.80	6.70	-0.01 (0.11)	
		20/18	Oral	Letter fluency	Score	11.21	1.45	40.20	10.70	-3.82 (0.32)	O
Baladzhaeva and Laufer (2018)	1	21/37	Oral	Letter fluency	Score	11.28	3.70	12.49	4.99	-0.26 (0.08)	
		21/37	Oral	Category fluency	Score	20.35	5.62	20.38	6.17	-0.00 (0.07)	
	2	30/37	Oral	Letter fluency	Score	11.06	4.13	12.49	4.99	-0.31 (0.06)	
		30/37	Oral	Category fluency	Score	20.38	6.17	20.38	6.17	0.00 (0.06)	
Barbu et al. (2020)	1	28/30	Oral	Letter fluency	Score	142.00	26.32	117.70	33.49	0.79 (0.07)	
	2	28/21	Oral	Letter fluency	Score	142.00	26.32	112.10	22.75	1.18 (0.10)	
Baus et al. (2020)	1	17/17	Oral	Picture naming	Reaction times	760.00	86.59	843.00	65.97	1.05 (0.14)	R
		17/17	Oral	Picture naming	Percent correct	87.00	8.25	92.00	4.12	-0.75 (0.13)	
		17/17	Oral	Picture naming	Reaction times	818.00	94.83	885.00	86.59	0.72 (0.13)	R
		17/17	Oral	Picture naming	Percent correct	85.00	8.25	85.00	4.12	0.00 (0.12)	
	2	17/19	Oral	Picture naming	Reaction times	760.00	86.59	857.00	78.46	1.15 (0.13)	R
		17/19	Oral	Picture naming	Percent correct	87.00	8.25	87.00	8.72	0.00 (0.11)	
		17/19	Oral	Picture naming	Reaction times	818.00	94.83	934.00	100.25	1.16 (0.13)	R
		17/19	Oral	Picture naming	Percent correct	85.00	8.25	78.00	8.72	0.81 (0.12)	
Bellegarda and Macizo (2021)	1	24/23	Oral	Picture naming	Percent correct	94.51	56.15	90.94	53.89	0.06 (0.09)	
		24/23	Oral	Picture naming	Reaction times	812.90	156.30	937.73	154.58	0.79 (0.09)	R
Bennett and Verney (2019)	1	30/99	Oral	Letter fluency	Score	12.07	4.56	10.85	3.78	0.31 (0.04)	
		30/99	Oral	Letter fluency	Score	10.77	2.64	9.17	3.69	0.46 (0.04)	
		30/99	Oral	Letter fluency	Score	14.20	4.62	12.35	4.49	0.41 (0.04)	
		30/99	Oral	Letter fluency	Score	13.50	3.24	11.37	3.78	0.58 (0.04)	
		30/99	Oral	Letter fluency	Score	12.43	3.41	11.27	3.66	0.32 (0.04)	
		30/99	Oral	Letter fluency	Score	12.93	3.87	10.94	3.61	0.54 (0.04)	
		30/99	Oral	Letter fluency	Score	12.80	3.75	11.30	3.49	0.42 (0.04)	
		30/99	Oral	Letter fluency	Score	11.37	3.69	10.40	3.86	0.25 (0.04)	
		30/99	Oral	Letter fluency	Score	11.70	3.16	10.40	3.65	0.36 (0.04)	
		30/99	Oral	Letter fluency	Score	12.17	3.12	10.94	3.94	0.32 (0.04)	
		30/99	Oral	Letter fluency	Score	37.10	10.05	32.43	10.21	0.46 (0.04)	
		30/99	Oral	Letter fluency	Score	38.90	8.76	33.64	10.01	0.54 (0.04)	
		30/99	Oral	Letter fluency	Score	35.87	8.71	32.24	9.56	0.39 (0.04)	
		30/99	Oral	Letter fluency	Score	36.00	8.55	32.58	10.22	0.34 (0.04)	
Berroir et al. (2017)	1	10/10	Oral	Letter fluency	Score	42.00	10.90	47.90	19.40	-0.36 (0.20)	
		10/10	Oral	Category fluency	Score	43.70	7.90	48.60	10.80	-0.50 (0.21)	
		10/10	Oral	Picture naming	Score	13.10	1.50	13.20	1.40	-0.07 (0.20)	
Bialystok et al. (2008)	1	24/24	Oral	Picture naming	Score	26.90	2.20	24.00	4.60	0.79 (0.09)	
		24/24	Oral	Picture naming	Score	25.50	3.20	20.40	4.30	1.32 (0.10)	

Study	Sample	N (m/b)	Domain	Task	Data type	M	SD	M	SD	Hedges' g	Notes
		24/24	Oral	Category fluency	Score	23.30	5.40	21.30	3.80	0.42 (0.09)	
		24/24	Oral	Letter fluency	Score	49.80	7.40	42.40	11.10	0.77 (0.09)	
	2	24/24	Oral	Picture naming	Score	27.10	2.30	23.00	3.00	1.51 (0.11)	
		24/24	Oral	Picture naming	Score	26.50	2.90	22.50	3.30	1.27 (0.10)	
		24/24	Oral	Category fluency	Score	20.10	3.90	15.70	5.40	0.92 (0.09)	
		24/24	Oral	Letter fluency	Score	45.40	7.70	34.90	13.90	0.92 (0.09)	
Bialystok et al. (2008)	1	24/24	Oral	Picture naming	Score	26.90	2.20	24.00	4.60	0.79 (0.09)	
		24/24	Oral	Category fluency	Score	23.30	5.40	21.30	3.80	0.42 (0.09)	
		24/24	Oral	Letter fluency	Score	16.60	2.50	14.10	3.70	0.78 (0.09)	
	2	16/50	Oral	Letter fluency	Score	12.90	3.90	13.80	4.20	-0.22 (0.08)	
		16/50	Oral	Category fluency	Score	20.00	4.80	19.20	5.50	0.15 (0.08)	
		16/49	Oral	Picture naming	Standardized scores	96.60	11.40	87.60	16.90	0.56 (0.09)	
Bice and Kroll (2021)	1	29/25	Oral	Category fluency	Score	13.70	2.61	12.96	2.23	0.30 (0.08)	
Blumenfeld and Marian (2014)	1	30/30		Letter fluency	Score	13.90	3.40	13.30	2.70	0.19 (0.07)	
		30/30		Category fluency	Score	16.70	4.50	16.40	3.00	0.08 (0.07)	
	2	60/60		Letter fluency	Score	14.00	3.40	12.60	3.30	0.42 (0.03)	
		60/60		Category fluency	Score	15.60	2.80	14.20	3.40	0.45 (0.03)	
Blumenfeld et al. (2016)	1	24/25	Oral	Letter fluency	Score	14.41	4.75	12.20	4.15	0.49 (0.08)	
		24/25	Oral	Letter fluency	Score	13.43	5.63	13.50	4.55	-0.01 (0.08)	
		24/25	Oral	Category fluency	Score	18.10	4.36	16.60	4.80	0.32 (0.08)	
		24/25	Oral	Category fluency	Score	14.44	5.63	15.00	4.95	-0.10 (0.08)	
Bogulski et al. (2015)	1	27/30	Oral	Letter fluency	Score	23.30	6.80	26.70	9.30	-0.41 (0.07)	
		27/30	Oral	Category fluency	Score	37.00	8.80	39.40	7.60	-0.29 (0.07)	
Bogulski et al. (2019)	1	21/22	Oral	Picture naming	Reaction times	774.70	126.94	764.51	115.34	-0.08 (0.09)	R
	2	21/19	Oral	Picture naming	Reaction times	773.80	132.44	922.34	155.87	1.01 (0.11)	R
	3	21/18	Oral	Picture naming	Reaction times	769.30	115.02	1055.00	110.31	2.48 (0.19)	R
Borodkin et al. (2016)	1	27/24	Oral	Category fluency	Score	23.07	5.13	17.70	4.95	1.05 (0.09)	
		27/24	Oral	Category fluency	Score	24.41	7.31	16.04	6.89	1.16 (0.09)	
Botezatu et al. (2021)	1	24/24	Oral	Picture naming	Percent correct	87.40	0.10	74.50	0.10	126.88 (181.98)	O
Bradley et al. (2013)	1	20/20	Oral	Picture naming	Score	54.05	2.93	48.30	4.53	1.48 (0.13)	
Broos et al. (2019)	1	54/54	Oral	Picture naming	Reaction times	826.61	223.67	949.71	288.71	0.47 (0.04)	R
Broos et al. (2021)	1	40/43	Oral	Picture naming	Reaction times	726.00	75.89	904.00	91.80	2.09 (0.08)	R
		40/43	Oral	Picture naming	Percent correct	75.00	12.65	48.00	13.11	2.07 (0.08)	
		40/43	Oral	Picture naming	Reaction times	715.00	69.57	919.00	111.48	2.16 (0.08)	R
		40/43	Oral	Picture naming	Percent correct	80.00	12.65	37.00	13.11	3.30 (0.12)	O
Clare et al. (2016)	1	49/49	Oral	Picture naming	Score	14.49	0.98	13.94	1.33	0.47 (0.04)	
Claussenius-Kalman et al. (2020)	1	145/121	Oral	Picture naming	Percent correct	81.37	7.50	73.82	7.65	0.99 (0.02)	
	2	145/68	Oral	Picture naming	Percent correct	81.37	7.50	68.43	10.32	1.52 (0.03)	
Costa et al. (2000)	1	21/21	Oral	Picture naming	Reaction times	690.00	76.00	718.00	66.00	0.39 (0.10)	R
		21/21	Oral	Picture naming	Reaction times	677.00	73.00	739.00	61.00	0.90 (0.11)	R
Costa et al. (2003)	1	10/10	Oral	Picture naming	Reaction times	638.00	75.00	800.00	69.00	2.15 (0.34)	R
		10/10	Oral	Picture naming	Reaction times	622.00	75.00	779.00	67.00	2.11 (0.34)	R
	2	10/10	Oral	Picture naming	Reaction times	638.00	75.00	681.00	90.00	0.50 (0.21)	R
		10/10	Oral	Picture naming	Reaction times	622.00	75.00	654.00	74.00	0.41 (0.21)	R
	3	24/24	Oral	Picture naming	Reaction times	713.00	71.00	728.00	76.00	0.20 (0.08)	R
		24/24	Oral	Picture naming	Reaction times	677.00	62.00	692.00	81.00	0.20 (0.08)	R
	4	24/24	Oral	Picture naming	Reaction times	713.00	71.00	782.00	91.00	0.83 (0.09)	R
		24/24	Oral	Picture naming	Reaction times	677.00	62.00	735.00	78.00	0.81 (0.09)	R
	5	10/10	Oral	Picture naming	Reaction times	657.00	100.00	845.00	62.00	2.16 (0.34)	R

Study	Sample	N (m/b)	Domain	Task	Data type	M	SD	M	SD	Hedges' g	Notes
		10/10	Oral	Picture naming	Reaction times	642.00	102.00	797.00	70.00	1.70 (0.29)	R
de Bruin et al. (2016)	1	24/28	Oral	Category fluency	Score	19.75	4.80	16.04	4.86	0.76 (0.08)	
		24/28	Oral	Letter fluency	Score	19.75	4.80	17.88	5.47	0.36 (0.08)	
	2	24/24	Oral	Category fluency	Score	14.42	5.91	14.33	5.57	0.02 (0.08)	
		24/24	Oral	Letter fluency	Score	14.42	5.91	13.14	4.97	0.23 (0.08)	
Degani and Tokowicz (2013)	1	30/30		Picture naming	Reaction times	909.80	121.90	1044.75	153.98	0.96 (0.07)	R
		30/30		Picture naming	Percent correct	93.00	5.00	82.00	13.00	1.10 (0.08)	
	2	30/30		Picture naming	Reaction times	909.80	121.90	880.83	125.78	-0.23 (0.07)	R
		30/30		Picture naming	Percent correct	93.00	5.00	96.00	3.00	-0.72 (0.07)	
Egan et al. (2019)	1	49/48	Oral	Letter fluency	Score	12.73	2.56	13.19	2.34	-0.19 (0.04)	
Emmorey et al. (2013)	1	24/18	Oral	Picture naming	Reaction times	881.70	316.96	1081.20	254.56	0.67 (0.10)	R
		24/18	Oral	Picture naming	Percent incorrect	4.00	4.90	2.95	4.24	-0.22 (0.10)	R
		24/18	Oral	Picture naming	Reaction times	915.80	446.79	1231.80	364.02	0.75 (0.10)	R
		24/18	Oral	Picture naming	Percent incorrect	8.97	9.94	12.95	8.70	0.41 (0.10)	R
	2	24/18	Oral	Picture naming	Reaction times	881.70	316.96	1067.00	158.67	0.69 (0.10)	R
		24/18	Oral	Picture naming	Percent incorrect	4.00	4.90	4.97	4.37	0.20 (0.10)	R
		24/18	Oral	Picture naming	Reaction times	915.80	446.79	1290.30	159.10	1.04 (0.11)	R
		24/18	Oral	Picture naming	Percent incorrect	9.00	9.80	14.95	4.45	0.73 (0.10)	R
	3	21/20	Oral	Picture naming	Reaction times	966.00	215.38	993.00	205.72	0.13 (0.10)	R
		21/20	Oral	Picture naming	Percent incorrect	4.96	4.77	2.95	4.70	-0.42 (0.10)	R
		21/20	Oral	Picture naming	Reaction times	1030.00	197.05	1018.00	178.89	-0.06 (0.10)	R
		21/20	Oral	Picture naming	Percent incorrect	8.96	4.77	4.96	4.65	-0.83 (0.11)	R
	4	21/20	Oral	Picture naming	Reaction times	966.00	215.38	902.00	196.77	-0.30 (0.10)	R
		21/20	Oral	Picture naming	Percent incorrect	4.96	4.77	4.00	4.47	-0.20 (0.10)	R
		21/20	Oral	Picture naming	Reaction times	1030.00	197.05	958.00	169.94	-0.38 (0.10)	R
		21/20	Oral	Picture naming	Percent incorrect	8.96	4.77	8.00	4.47	-0.20 (0.10)	R
Estanga et al. (2017)	1	100/97	Oral	Category fluency	Score	22.38	5.72	24.32	5.96	-0.33 (0.02)	
		100/97	Oral	Letter fluency	Score	16.50	5.03	18.69	5.66	-0.41 (0.02)	
		100/97	Oral	Picture naming	Score	54.70	3.84	55.04	3.88	-0.09 (0.02)	
	2	100/81	Oral	Category fluency	Score	22.38	5.72	23.94	6.12	-0.26 (0.02)	
		100/81	Oral	Letter fluency	Score	16.50	5.03	17.98	4.27	-0.31 (0.02)	
		100/81	Oral	Picture naming	Score	54.70	3.84	54.56	3.95	0.04 (0.02)	
Fernandez et al. (2013)	1	15/13	Oral	Picture naming	Score	33.93	4.08	31.31	3.61	0.66 (0.15)	
Filippi et al. (2022)	1	86/84	Oral	Letter fluency	Score	48.20	15.10	43.30	12.40	0.35 (0.02)	
		86/84	Oral	Category fluency	Score	76.20	13.00	69.80	16.60	0.43 (0.02)	
Francis et al. (2018)	1	128/64	Oral	Picture naming	Score	16.10	4.70	14.20	3.30	0.44 (0.02)	
	2	128/64	Oral	Picture naming	Score	16.10	4.70	10.40	1.50	1.44 (0.03)	
Friesen et al. (2015)	1	20/20	Oral	Category fluency	Score	19.90	4.60	19.00	5.70	0.17 (0.10)	
		20/20	Oral	Category fluency	Reaction times	22.90	4.20	23.20	6.00	0.06 (0.10)	R
		20/20	Oral	Letter fluency	Score	11.80	3.50	15.30	4.00	-0.91 (0.11)	
		20/20	Oral	Letter fluency	Reaction times	24.10	3.60	26.60	2.90	0.75 (0.11)	R
	2	20/21	Oral	Category fluency	Score	17.00	3.00	17.90	3.50	-0.27 (0.10)	
		20/21	Oral	Category fluency	Reaction times	20.60	3.30	21.80	3.60	0.34 (0.10)	R
		20/21	Oral	Letter fluency	Score	12.90	3.50	14.70	4.60	-0.43 (0.10)	
		20/21	Oral	Letter fluency	Reaction times	23.20	3.90	24.30	2.60	0.33 (0.10)	R
Giezen and Emmorey (2017)	1	19/19	Oral	Letter fluency	Score	15.80	5.30	12.80	5.30	0.55 (0.11)	
		19/19	Oral	Letter fluency	Score	13.20	3.80	11.80	3.90	0.36 (0.11)	
		19/19	Oral	Letter fluency	Score	18.40	5.70	15.80	5.20	0.47 (0.11)	
		19/19	Oral	Letter fluency	Score	11.90	4.50	10.00	3.50	0.46 (0.11)	

Study	Sample	N (m/b)	Domain	Task	Data type	M	SD	M	SD	Hedges' g	Notes
		19/19	Oral	Letter fluency	Score	18.10	5.10	13.40	4.70	0.94 (0.12)	
		19/19	Oral	Letter fluency	Score	16.20	4.10	12.90	4.10	0.79 (0.11)	
Gollan and Brown (2006)	1	36/36	Oral	Picture naming	Percent correct	27.00	14.00	9.00	12.00	1.37 (0.07)	
		36/36	Oral	Picture naming	Percent correct	54.00	11.00	33.00	16.00	1.51 (0.07)	
		36/36	Oral	Picture naming	Percent correct	79.00	9.00	64.00	15.00	1.20 (0.07)	
Gollan et al. (2002)	1	30/30	Oral	Letter fluency	Score	13.07	4.01	9.80	3.63	0.84 (0.07)	
		30/30	Oral	Letter fluency	Score	11.73	3.19	10.13	2.64	0.54 (0.07)	
		30/30	Oral	Letter fluency	Score	12.33	3.12	10.67	3.20	0.52 (0.07)	
		30/30	Oral	Letter fluency	Score	12.37	3.16	12.13	2.67	0.08 (0.07)	
		30/30	Oral	Letter fluency	Score	13.73	3.40	12.67	4.55	0.26 (0.07)	
		30/30	Oral	Letter fluency	Score	13.47	4.20	12.93	3.53	0.14 (0.07)	
		30/30	Oral	Letter fluency	Score	13.27	2.90	13.00	2.36	0.10 (0.07)	
		30/30	Oral	Letter fluency	Score	15.60	3.44	13.80	2.65	0.58 (0.07)	
		30/30	Oral	Letter fluency	Score	16.73	3.69	13.87	4.03	0.73 (0.07)	
		30/30	Oral	Letter fluency	Score	14.80	3.68	14.53	2.56	0.08 (0.07)	
		30/30	Oral	Category fluency	Score	7.30	2.58	6.53	1.68	0.35 (0.07)	
		30/30	Oral	Category fluency	Score	11.00	4.07	8.00	4.36	0.70 (0.07)	
		30/30	Oral	Category fluency	Score	10.43	2.06	8.73	3.20	0.62 (0.07)	
		30/30	Oral	Category fluency	Score	14.50	3.10	10.27	1.91	1.62 (0.09)	
		30/30	Oral	Category fluency	Score	12.73	2.92	10.47	2.36	0.84 (0.07)	
		30/30	Oral	Category fluency	Score	13.57	2.88	11.67	3.77	0.56 (0.07)	
		30/30	Oral	Category fluency	Score	15.07	2.70	11.87	1.64	1.41 (0.08)	
		30/30	Oral	Category fluency	Score	16.03	3.87	12.00	2.54	1.22 (0.08)	
		30/30	Oral	Category fluency	Score	15.60	2.59	13.67	2.97	0.68 (0.07)	
		30/30	Oral	Category fluency	Score	15.50	2.67	14.00	2.59	0.56 (0.07)	
		30/30	Oral	Category fluency	Score	22.83	7.58	16.00	8.43	0.84 (0.07)	
		30/30	Oral	Category fluency	Score	20.47	4.52	16.80	4.72	0.78 (0.07)	
		30/30	Oral	Letter fluency	Score	8.13	2.71	6.17	2.15	0.79 (0.07)	
		30/30	Oral	Letter fluency	Score	10.07	2.78	8.67	2.14	0.56 (0.07)	
Gollan et al. (2005)	1	31/31	Oral	Picture naming	Reaction times	903.77	112.00	987.77	140.00	0.65 (0.07)	R
		31/31	Oral	Picture naming	Percent incorrect	8.70	5.90	10.50	6.00	0.30 (0.07)	R
	2	36/36	Oral	Picture naming	Reaction times	865.20	105.00	937.80	107.00	0.68 (0.06)	R
		36/36	Oral	Picture naming	Percent incorrect	5.30	5.80	12.90	5.60	1.32 (0.07)	R
Gollan et al. (2005)	1	28/28	Oral	Picture naming	Percent correct	0.66	0.12	0.43	0.15	1.67 (0.10)	
Gollan et al. (2008)	1	57/57	Oral	Picture naming	Reaction times	848.00	166.10	931.00	218.95	0.42 (0.04)	R
		57/57	Oral	Picture naming	Percent incorrect	3.10	3.02	3.00	3.02	-0.03 (0.04)	R
		57/57	Oral	Picture naming	Reaction times	928.00	226.50	1080.00	241.59	0.64 (0.04)	R
		57/57	Oral	Picture naming	Percent incorrect	5.00	6.79	5.00	6.79	0.00 (0.04)	R
	2	21/20	Oral	Picture naming	Reaction times	1075.00	137.48	1240.00	147.58	1.14 (0.11)	R
		21/20	Oral	Picture naming	Percent incorrect	4.00	2.29	7.40	2.24	1.47 (0.13)	R
		21/20	Oral	Picture naming	Reaction times	1210.00	197.05	1477.00	187.83	1.36 (0.12)	R
		21/20	Oral	Picture naming	Percent incorrect	7.20	2.75	10.00	4.47	0.74 (0.11)	R
Gollan et al. (2011)	1	50/50	Oral	Picture naming	Reaction times	859.35	139.65	971.00	127.28	0.83 (0.04)	R
		50/50	Oral	Picture naming	Reaction times	924.60	133.64	1095.50	115.26	1.36 (0.05)	R
	2	50/45	Oral	Picture naming	Reaction times	859.35	139.65	1090.40	80.50	1.98 (0.06)	R
		50/45	Oral	Picture naming	Reaction times	924.60	133.64	1296.50	86.54	3.24 (0.10)	R O
Gollan et al. (2012)	1	36/35	Oral	Picture naming	Percent correct	0.90	0.05	0.81	0.10	1.06 (0.06)	
		36/35	Oral	Picture naming	Percent correct	0.95	0.03	0.91	0.04	1.30 (0.07)	
	2	36/7	Oral	Picture naming	Percent correct	0.90	0.05	0.83	0.10	1.09 (0.19)	

Study	Sample	N (m/b)	Domain	Task	Data type	M	SD	M	SD	Hedges' g	Notes
		36/7	Oral	Picture naming	Percent correct	0.95	0.03	0.91	0.05	1.33 (0.19)	
	3	36/10	Oral	Picture naming	Percent correct	0.90	0.05	0.69	0.10	3.12 (0.24)	O
		36/10	Oral	Picture naming	Percent correct	0.95	0.03	0.83	0.06	3.36 (0.26)	O
Hirsh et al. (2003)	1	23/40	Oral	Picture naming	Reaction times	852.00	159.00	1397.00	312.00	2.02 (0.10)	R
Ivanova et al. (2013)	1	44/11	Oral	Picture naming	Percent correct	0.96	0.04	0.64	0.22	3.09 (0.21)	O
		44/11	Oral	Picture naming	Percent correct	0.94	0.07	0.47	0.23	3.92 (0.26)	O
Johns et al. (2016)	1	21/21	Oral	Picture naming	Score	53.33	3.28	50.18	5.60	0.67 (0.10)	
		21/21	Oral	Letter fluency	Score	40.38	14.06	37.04	9.86	0.27 (0.10)	
		21/21	Oral	Category fluency	Score	26.29	5.32	24.57	5.69	0.31 (0.10)	
	2	16/28	Oral	Picture naming	Score	55.87	2.92	51.71	4.81	0.97 (0.11)	
		16/28	Oral	Letter fluency	Score	43.40	7.43	40.43	10.42	0.31 (0.10)	
		16/28	Oral	Category fluency	Score	21.47	5.87	18.81	4.64	0.51 (0.10)	
Kalia et al. (2014)	1	42/40	Oral	Picture naming	Score	116.21	12.65	112.33	14.06	0.29 (0.05)	
	2	42/23	Oral	Picture naming	Score	116.21	12.65	117.22	13.88	-0.08 (0.07)	
Kan and Sadagopan (2014)	1	10/10	Oral	Picture naming	Score	46.44	1.94	42.80	4.80	0.95 (0.23)	
Kasparian and Steinhauer (2016)	1	30/24	Oral	Category fluency	Score	23.40	5.50	21.50	3.90	0.39 (0.08)	
Kasparian and Steinhauer (2017)	1	30/24	Oral	Category fluency	Score	23.40	5.50	21.50	3.90	0.39 (0.08)	
Kaushanskaya and Marian (2009)	1	20/20		Picture naming	Percentile	89.85	20.00	81.66	20.60	0.40 (0.10)	
	2	20/20		Picture naming	Percentile	89.85	20.00	93.00	21.20	-0.15 (0.10)	
Kaushanskaya and Marian (2009)	1	24/24	Oral	Picture naming	Percentile	90.34	4.51	81.66	4.96	1.80 (0.12)	
Kaushanskaya et al. (2011)	1	30/30	Oral	Picture naming	Percentile	91.57	20.48	82.90	20.16	0.42 (0.07)	
Kharkhurin (2008)	1	46/103	Oral	Picture naming	Score	110.66	7.90	91.19	16.47	1.35 (0.04)	
Kharkhurin (2009)	1	37/34	Oral	Picture naming	Score	103.38	10.64	105.91	10.62	-0.24 (0.06)	
Kharkhurin (2010)	1	47/103	Oral	Picture naming	Score	110.66	7.91	91.19	16.47	1.35 (0.04)	
Kharkhurin (2010)	1	47/103	Oral	Picture naming	Score	110.66	7.91	91.19	16.47	1.35 (0.04)	
	2	37/34	Oral	Picture naming	Score	103.38	10.64	105.91	10.62	-0.24 (0.06)	
Kharkhurin (2017)	1	28/58	Written	Picture naming	Score	94.36	13.83	99.57	9.54	-0.47 (0.05)	
Kousaie et al. (2014)	1	30/51	Oral	Picture naming	Score	49.40	4.55	46.92	9.85	0.30 (0.05)	
		30/51	Oral	Letter fluency	Score	38.27	8.26	37.24	10.26	0.11 (0.05)	
		30/51	Oral	Category fluency	Score	23.00	5.07	23.96	5.77	-0.17 (0.05)	
	2	40/51	Oral	Picture naming	Score	53.20	3.44	46.92	9.85	0.81 (0.05)	
		40/51	Oral	Letter fluency	Score	39.95	12.83	37.24	10.26	0.23 (0.04)	
		40/51	Oral	Category fluency	Score	25.02	5.90	23.96	5.77	0.18 (0.04)	
	3	30/36	Oral	Picture naming	Score	46.93	5.92	48.56	6.00	-0.27 (0.06)	
		30/36	Oral	Letter fluency	Score	36.37	10.13	37.00	13.26	-0.05 (0.06)	
		30/36	Oral	Category fluency	Score	18.27	4.62	19.17	6.41	-0.16 (0.06)	
	4	31/36	Oral	Picture naming	Score	55.63	3.16	48.56	6.00	1.43 (0.08)	
		31/36	Oral	Letter fluency	Score	44.10	10.00	37.00	13.26	0.59 (0.06)	
		31/36	Oral	Category fluency	Score	20.68	4.58	19.17	6.41	0.26 (0.06)	
Kovelman et al. (2009)	1	20/5	Oral	Picture naming	Percent correct	89.80	0.05	90.00	0.05	-3.87 (0.60)	O
	2	7/5	Oral	Picture naming	Percent correct	95.20	0.03	88.50	0.10	91.75 (509.16)	O
Kreiner and Degani (2015)	1	24/24	Oral	Picture naming	Percent correct	98.12	1.67	90.75	4.99	1.95 (0.13)	
	2	24/24	Oral	Picture naming	Percent correct	98.12	1.67	96.84	2.03	0.68 (0.09)	
Li et al. (2013)	1	11/15	Oral	Picture naming	Percent correct	98.72	2.00	87.18	7.50	1.90 (0.24)	
		11/15	Oral	Picture naming	Reaction times	731.00	47.56	898.00	59.60	2.95 (0.35)	R
Li et al. (2015)	1	13/14	Oral	Picture naming	Reaction times	785.00	36.00	793.00	110.00	0.09 (0.15)	R
Li et al. (2017)	1	32/32	Oral	Picture naming	Score	65.00	2.00	60.20	3.80	1.56 (0.08)	
	2	32/32	Oral	Picture naming	Score	65.00	2.00	60.10	7.60	0.87 (0.07)	
Lin and Lin (2016)	1	24/67	Oral	Picture naming	Score	41.70	3.90	25.00	6.20	2.91 (0.11)	

Study	Sample	N (m/b)	Domain	Task	Data type	M	SD	M	SD	Hedges' g	Notes
	2	24/46	Oral	Picture naming	Score	41.70	3.90	39.30	4.00	0.60 (0.07)	
Ljungberg et al. (2013)	1	3/10	Oral	Letter fluency	Score	9.33	4.04	14.70	6.15	-0.86 (0.47)	
		3/10	Oral	Category fluency	Score	3.33	0.58	4.90	2.64	-0.61 (0.45)	
	2	6/34	Oral	Letter fluency	Score	9.50	3.39	14.80	4.21	-1.26 (0.22)	
		6/34	Oral	Category fluency	Score	4.17	2.32	5.76	2.54	-0.62 (0.20)	
	3	6/23	Oral	Letter fluency	Score	8.83	2.99	15.91	4.36	-1.66 (0.26)	
		6/23	Oral	Category fluency	Score	3.33	2.50	5.74	2.49	-0.94 (0.23)	
	4	13/16	Oral	Letter fluency	Score	11.85	2.54	15.75	5.31	-0.88 (0.15)	
		13/16	Oral	Category fluency	Score	5.69	2.02	5.63	2.42	0.03 (0.14)	
	5	16/6	Oral	Letter fluency	Score	8.94	4.20	13.00	6.48	-0.80 (0.25)	
		16/6	Oral	Category fluency	Score	4.81	1.91	4.17	2.14	0.31 (0.23)	
	6	16/6	Oral	Letter fluency	Score	10.19	3.83	18.17	4.02	-1.98 (0.34)	
		16/6	Oral	Category fluency	Score	4.31	2.36	5.00	1.26	-0.31 (0.23)	
	7	6/6	Oral	Letter fluency	Score	9.50	5.24	14.83	5.23	-0.94 (0.39)	
		6/6	Oral	Category fluency	Score	3.17	0.75	5.67	3.20	-0.99 (0.39)	
	8	8/3	Oral	Letter fluency	Score	10.38	3.02	15.33	5.77	-1.19 (0.56)	O
		8/3	Oral	Category fluency	Score	5.25	2.49	5.00	1.73	0.10 (0.46)	
Ljungberg et al. (2020)	1	26/26	Oral	Letter fluency	Score	9.00	3.66	11.15	3.79	-0.57 (0.08)	
		26/26	Oral	Category fluency	Score	4.35	1.87	4.57	2.10	-0.11 (0.08)	
	2	26/26	Oral	Letter fluency	Score	9.00	3.66	12.27	4.47	-0.79 (0.08)	
		26/26	Oral	Category fluency	Score	4.35	1.87	4.23	2.32	0.06 (0.08)	
Luo et al. (2010)	1	20/20	Oral	Picture naming	Standardized scores	98.90	8.40	97.30	13.00	0.14 (0.10)	
		20/20	Oral	Letter fluency	Score	11.80	3.50	15.40	4.00	-0.94 (0.11)	
		20/20	Oral	Category fluency	Score	21.10	4.20	20.00	6.10	0.21 (0.10)	
	2	20/20	Oral	Picture naming	Standardized scores	98.90	8.40	85.80	10.20	1.37 (0.13)	
		20/20	Oral	Letter fluency	Score	11.80	3.50	11.20	3.30	0.17 (0.10)	
		20/20	Oral	Category fluency	Score	21.10	4.20	19.10	3.30	0.52 (0.10)	
Massa et al. (2020)	1	16/16	Oral	Letter fluency	Score	25.70	6.30	26.20	4.50	-0.09 (0.13)	
		16/16	Oral	Category fluency	Score	15.90	5.60	17.80	5.00	-0.35 (0.13)	
	2	16/16	Oral	Letter fluency	Score	24.60	6.00	26.90	5.10	-0.40 (0.13)	
		16/16	Oral	Category fluency	Score	15.40	4.50	20.80	5.40	-1.06 (0.14)	
Milman et al. (2018)	1	16/16	Oral	Picture naming	Score	55.80	3.00	32.30	13.40	2.36 (0.22)	
	2	14/14	Oral	Picture naming	Score	55.40	4.30	40.40	8.50	2.16 (0.24)	
Misdraji-Hammond et al. (2015)	1	52/37	Oral	Picture naming	Score	46.69	3.61	42.49	6.97	0.79 (0.05)	
Mizrahi et al. (2021)	1	32/31	Oral	Picture naming	Score	64.30	2.20	60.50	3.40	1.32 (0.08)	
	2	32/32	Oral	Picture naming	Score	64.30	2.20	61.10	3.30	1.13 (0.07)	
	3	32/32	Oral	Picture naming	Score	64.30	2.20	60.20	3.00	1.54 (0.08)	
	4	32/32	Oral	Picture naming	Score	64.30	2.20	61.70	3.10	0.96 (0.07)	
	5	32/31	Oral	Picture naming	Score	64.70	2.10	60.50	3.40	1.47 (0.08)	
	6	32/32	Oral	Picture naming	Score	64.70	2.10	61.10	3.30	1.29 (0.08)	
	7	32/32	Oral	Picture naming	Score	64.70	2.10	60.20	3.00	1.72 (0.09)	
	8	32/32	Oral	Picture naming	Score	64.70	2.10	61.70	3.10	1.12 (0.07)	
Mor et al. (2015)	1	20/20	Oral	Category fluency	Score	17.90	4.12	18.68	4.01	-0.19 (0.10)	
		20/20	Oral	Letter fluency	Score	13.40	3.89	14.10	3.77	-0.18 (0.10)	
Morrison and Taler (2020)	1	26/28	Oral	Picture naming	Score	53.04	3.34	50.18	7.61	0.47 (0.08)	
		26/28	Oral	Letter fluency	Score	39.62	9.08	41.79	13.94	-0.18 (0.07)	
		26/28	Oral	Category fluency	Score	24.00	5.34	22.32	6.09	0.29 (0.08)	
	2	28/29	Oral	Picture naming	Score	52.36	4.28	49.48	5.17	0.60 (0.07)	
		28/29	Oral	Letter fluency	Score	42.04	11.18	39.86	10.28	0.20 (0.07)	

Study	Sample	N (m/b)	Domain	Task	Data type	M	SD	M	SD	Hedges' g	Notes
		28/29	Oral	Category fluency	Score	19.64	5.12	17.90	3.59	0.39 (0.07)	
Navarro-Torres et al. (2019)	1	23/24	Oral	Category fluency	Score	58.22	8.38	45.04	8.61	1.52 (0.11)	
Olabarrieta-Landa Laiene et al. (2019)	1	87/139	Oral	Letter fluency	Score	11.85	4.88	13.70	4.83	-0.38 (0.02)	
		87/139	Oral	Letter fluency	Score	12.09	4.04	12.11	4.53	-0.00 (0.02)	
		87/139	Oral	Letter fluency	Score	12.59	4.81	14.17	4.80	-0.33 (0.02)	
		87/139	Oral	Letter fluency	Score	13.46	4.84	14.67	4.69	-0.25 (0.02)	
		87/139	Oral	Letter fluency	Score	13.22	4.69	14.47	4.77	-0.26 (0.02)	
		87/139	Oral	Letter fluency	Score	15.16	4.82	15.99	4.23	-0.19 (0.02)	
		87/139	Oral	Letter fluency	Score	10.15	4.00	11.59	4.79	-0.32 (0.02)	
		87/139	Oral	Letter fluency	Score	9.41	4.11	10.09	4.11	-0.16 (0.02)	
	2	87/130	Oral	Letter fluency	Score	11.85	4.88	11.96	4.24	-0.02 (0.02)	
		87/130	Oral	Letter fluency	Score	12.09	4.04	12.52	4.45	-0.10 (0.02)	
		87/130	Oral	Letter fluency	Score	12.59	4.81	13.78	4.44	-0.26 (0.02)	
		87/130	Oral	Letter fluency	Score	13.46	4.84	13.70	4.19	-0.05 (0.02)	
		87/130	Oral	Letter fluency	Score	13.22	4.69	13.75	4.18	-0.12 (0.02)	
		87/130	Oral	Letter fluency	Score	15.16	4.82	15.78	4.88	-0.13 (0.02)	
		87/130	Oral	Letter fluency	Score	10.15	4.00	10.95	4.03	-0.20 (0.02)	
		87/130	Oral	Letter fluency	Score	9.41	4.11	10.41	3.78	-0.25 (0.02)	
Olsen et al. (2015)	1	14/13	Oral	Letter fluency	Score	13.60	4.00	15.40	5.70	-0.36 (0.15)	
		14/13	Oral	Category fluency	Score	18.40	3.30	20.10	3.10	-0.51 (0.15)	
Oschwald et al. (2018)	1	25/26	Written	Category fluency	Score	108.28	23.26	107.35	20.33	0.04 (0.08)	
	2	25/24	Written	Category fluency	Score	108.28	23.26	99.40	17.02	0.43 (0.08)	
Paap et al. (2017)	1	108/122	Oral	Category fluency	Score	12.00	3.64	11.10	3.87	0.24 (0.02)	
		108/122	Oral	Category fluency	Score	13.30	3.33	12.30	3.64	0.28 (0.02)	
		108/122	Oral	Letter fluency	Score	12.50	4.36	11.30	3.64	0.30 (0.02)	
		108/122	Oral	Letter fluency	Score	15.20	4.47	13.40	3.98	0.43 (0.02)	
Palomar-García et al. (2015)	1	21/23	Oral	Picture naming	Reaction times	1117.00	196.00	1267.00	217.00	0.71 (0.10)	R
Paplikar et al. (2021)	1	74/67	Oral	Picture naming	Score	89.30	1.90	88.50	4.30	0.24 (0.03)	
	2	74/67	Oral	Category fluency	Score	10.20	3.40	11.10	3.20	-0.27 (0.03)	
	3	32/32	Oral	Picture naming	Score	79.40	7.20	82.90	6.30	-0.51 (0.06)	
	4	32/32	Oral	Category fluency	Score	15.60	3.60	17.60	3.60	-0.55 (0.06)	
	5	80/80	Oral	Picture naming	Score	80.90	5.00	79.80	6.10	0.20 (0.03)	
	6	80/80	Oral	Category fluency	Score	14.00	3.20	14.40	3.10	-0.13 (0.03)	
	7	41/42	Oral	Picture naming	Score	80.80	5.50	83.20	4.80	-0.46 (0.05)	
	8	41/42	Oral	Category fluency	Score	12.20	3.20	12.20	3.90	0.00 (0.05)	
	9	40/42	Oral	Picture naming	Score	81.50	6.20	82.10	5.10	-0.10 (0.05)	
	10	40/42	Oral	Category fluency	Score	14.60	2.60	13.20	2.60	0.53 (0.05)	
Patra et al. (2020)	1	25/25	Oral	Category fluency	Score	21.50	3.50	19.80	3.10	0.51 (0.08)	
		25/25	Oral	Letter fluency	Score	15.80	3.50	17.80	3.50	-0.56 (0.08)	
Pelham and Abrams (2013)	1	30/20	Oral	Picture naming	Reaction times	761.60	72.30	848.20	106.20	0.98 (0.09)	R
	2	30/20	Oral	Picture naming	Reaction times	761.60	72.30	849.70	104.90	1.00 (0.09)	R
Peñaloza et al. (2019)	1	3/3	Oral	Picture naming	Percent correct	94.00	2.90	89.00	8.30	0.64 (0.76)	O
	2	3/25	Oral	Picture naming	Percent correct	94.00	2.90	75.50	16.20	1.15 (0.40)	
	3	2/3	Oral	Picture naming	Percent correct	90.00	2.80	51.50	40.50	0.84 (1.09)	O
	4	2/25	Oral	Picture naming	Percent correct	90.00	2.80	86.50	11.40	0.30 (0.54)	
Peristeri et al. (2018)	1	20/16	Oral	Picture naming	Percent incorrect	0.30	0.60	0.30	0.50	0.00 (0.11)	R
		20/16	Oral	Picture naming	Reaction times	482.00	87.00	494.00	54.00	0.16 (0.11)	R
	2	20/16	Oral	Picture naming	Percent incorrect	0.30	0.60	0.30	0.80	0.00 (0.11)	R
		20/16	Oral	Picture naming	Reaction times	482.00	87.00	519.00	59.00	0.48 (0.12)	R

Study	Sample	N (m/b)	Domain	Task	Data type	M	SD	M	SD	Hedges' g	Notes
Portocarrero et al. (2007)	1	39/39	Oral	Letter fluency	Score	36.90	10.10	35.00	7.50	0.21 (0.05)	
		39/39	Oral	Category fluency	Score	54.80	10.60	46.20	9.10	0.86 (0.06)	
		39/39	Oral	Picture naming	Standardized scores	107.30	12.30	94.90	15.30	0.88 (0.06)	
Prior and Gollan (2011)	1	47/41	Oral	Category fluency	Score	35.90	4.00	30.10	4.80	1.31 (0.06)	
	2	47/43	Oral	Category fluency	Score	35.90	4.00	35.20	3.30	0.19 (0.04)	
Pyers et al. (2009)	1	22/22	Oral	Picture naming	Score	40.18	4.14	35.41	4.09	1.14 (0.11)	
	2	22/11	Oral	Picture naming	Score	40.18	4.14	31.00	5.20	1.99 (0.20)	
Roberts et al. (2002)	1	42/32	Oral	Picture naming	Score	50.90	3.45	42.60	8.04	1.40 (0.07)	
	2	42/49	Oral	Picture naming	Score	50.90	3.45	39.50	7.43	1.90 (0.06)	
Rodriguez-Fornells et al. (2005)	1	11/11	Oral	Picture naming	Score	58.70	0.64	54.70	4.60	1.17 (0.22)	
Rosselli et al. (2000)	1	18/19	Oral	Picture naming	Score	51.10	4.10	52.90	6.10	-0.34 (0.11)	
		18/19	Oral	Category fluency	Score	16.70	3.80	14.50	3.80	0.57 (0.11)	
		18/19	Oral	Category fluency	Score	14.80	3.80	11.30	3.60	0.93 (0.12)	
		18/19	Oral	Letter fluency	Score	11.70	4.10	11.30	4.30	0.09 (0.11)	
		18/19	Oral	Letter fluency	Score	11.80	4.60	12.30	4.60	-0.11 (0.11)	
		18/19	Oral	Letter fluency	Score	11.40	3.80	11.60	5.40	-0.04 (0.11)	
	2	45/19	Oral	Picture naming	Score	54.90	4.80	52.40	7.10	0.44 (0.08)	
		45/19	Oral	Category fluency	Score	16.80	5.20	14.20	4.10	0.52 (0.08)	
		45/19	Oral	Category fluency	Score	13.70	3.60	11.60	3.20	0.59 (0.08)	
		45/19	Oral	Letter fluency	Score	12.90	5.40	12.50	5.00	0.07 (0.07)	
		45/19	Oral	Letter fluency	Score	10.70	5.10	10.70	5.40	0.00 (0.07)	
		45/19	Oral	Letter fluency	Score	13.80	5.40	12.40	3.90	0.28 (0.08)	
Rosselli et al. (2002)	1	45/19	Oral	Letter fluency	Score	12.90	5.40	12.50	5.00	0.07 (0.07)	
		45/19	Oral	Letter fluency	Score	10.70	5.10	10.70	5.40	0.00 (0.07)	
		45/19	Oral	Letter fluency	Score	13.80	5.40	12.40	3.90	0.28 (0.08)	
		45/19	Oral	Category fluency	Score	16.80	5.20	14.20	4.10	0.52 (0.08)	
	2	18/19	Oral	Letter fluency	Score	11.70	4.10	11.30	4.30	0.09 (0.11)	
		18/19	Oral	Letter fluency	Score	11.80	4.60	12.30	4.60	-0.11 (0.11)	
		18/19	Oral	Letter fluency	Score	11.40	3.80	11.60	5.40	-0.04 (0.11)	
		18/19	Oral	Category fluency	Score	16.70	3.80	14.50	3.80	0.57 (0.11)	
Rosselli et al. (2016)	1	20/20	Oral	Picture naming	Standardized scores	534.00	11.10	525.20	13.00	0.71 (0.11)	
	2	20/20	Oral	Picture naming	Standardized scores	534.00	11.10	496.60	25.80	1.85 (0.15)	
	3	20/34	Oral	Picture naming	Standardized scores	534.00	11.10	510.40	17.80	1.48 (0.10)	
	4	20/20	Oral	Picture naming	Standardized scores	509.80	21.60	525.20	13.00	-0.85 (0.11)	
	5	20/20	Oral	Picture naming	Standardized scores	509.80	21.60	496.60	25.80	0.54 (0.10)	
	6	20/34	Oral	Picture naming	Standardized scores	509.80	21.60	510.40	17.80	-0.03 (0.08)	
Runnqvist et al. (2013)	1	41/34	Oral	Picture naming	Percent correct	96.00	3.00	92.00	5.00	0.98 (0.06)	
	2	41/34	Oral	Picture naming	Percent correct	96.00	3.00	92.00	4.00	1.14 (0.06)	
Ryskin et al. (2014)	1	21/20	Oral	Picture naming	Reaction times	1715.91	268.25	1866.55	272.86	0.55 (0.10)	R
Sadat et al. (2012)	1	35/35	Oral	Picture naming	Reaction times	654.00	76.00	708.00	76.00	0.70 (0.06)	R
	2	35/35	Oral	Picture naming	Reaction times	688.00	92.00	741.00	91.00	0.57 (0.06)	R
Sadat et al. (2016)	1	30/30	Oral	Picture naming	Reaction times	910.00	118.00	947.00	92.00	0.35 (0.07)	R
	2	30/30	Oral	Picture naming	Reaction times	910.00	118.00	964.00	93.00	0.50 (0.07)	R
Salvatierra and Rosselli (2010)	1	66/67	Oral	Picture naming	Score	51.82	5.90	45.36	9.90	0.79 (0.03)	
	2	42/58	Oral	Picture naming	Score	52.02	10.40	44.07	9.10	0.82 (0.04)	
Sandoval et al. (2010)	1	30/24	Oral	Category fluency	Score	13.80	2.90	12.50	2.70	0.46 (0.08)	
		30/24	Oral	Category fluency	Reaction times	2.00	1.00	3.20	1.60	0.91 (0.08)	R
		30/24	Oral	Letter fluency	Score	7.00	1.00	6.30	1.10	0.66 (0.08)	
		30/24	Oral	Letter fluency	Reaction times	2.40	1.10	2.80	1.50	0.31 (0.08)	R

Study	Sample	N (m/b)	Domain	Task	Data type	M	SD	M	SD	Hedges' g	Notes
Sasisekaran and Weisberg (2013)	1	13/7	Oral	Picture naming	Score	170.50	5.80	165.40	6.50	0.81 (0.24)	
Savoie et al. (2019)	1	38/27	Oral	Picture naming	Score	43.70	8.03	40.78	5.96	0.40 (0.06)	
Schmid (2014)	1	53/53	Oral	Category fluency	Score	25.80	4.60	21.80	4.70	0.85 (0.04)	
	2	53/20	Oral	Category fluency	Score	25.80	4.60	22.90	4.70	0.62 (0.07)	
Schmidtke (2014)	1	21/17	Oral	Picture naming	Standardized scores	99.50	6.90	93.60	10.70	0.66 (0.11)	
	2	21/15	Oral	Picture naming	Standardized scores	99.50	6.90	78.50	9.20	2.59 (0.22)	
Schmidtke (2016)	1	53/48	Oral	Picture naming	Standardized scores	101.00	7.60	86.00	8.40	1.86 (0.06)	
Seçer (2016)	1	88/74	Oral	Letter fluency	Score	39.28	9.18	36.56	10.63	0.27 (0.03)	
Segal and Gollan (2018)	1	57/57	Oral	Picture naming	Score	65.10	2.20	60.70	3.20	1.59 (0.05)	
Sehyr et al. (2018)	1	25/24	Oral	Category fluency	Score	16.80	3.60	14.40	3.92	0.63 (0.09)	
	2	29/24	Oral	Category fluency	Score	18.00	3.07	20.10	3.92	-0.59 (0.08)	
	3	25/23	Oral	Category fluency	Score	16.80	3.60	15.50	3.84	0.34 (0.08)	
	4	29/23	Oral	Category fluency	Score	18.00	3.07	19.20	3.84	-0.34 (0.08)	
Sheppard et al. (2016)	1	41/47	Oral	Category fluency	Score	24.34	5.35	23.54	6.62	0.13 (0.05)	
		41/47	Oral	Letter fluency	Score	40.53	12.28	37.79	10.10	0.24 (0.05)	
		41/47	Oral	Picture naming	Score	53.59	3.57	49.47	6.27	0.79 (0.05)	
	2	30/47	Oral	Category fluency	Score	23.00	5.07	23.54	6.62	-0.09 (0.05)	
		30/47	Oral	Letter fluency	Score	38.27	8.26	37.79	10.10	0.05 (0.05)	
		30/47	Oral	Picture naming	Score	50.27	4.25	38.98	8.84	1.51 (0.07)	
	3	31/36	Oral	Category fluency	Score	20.68	4.58	19.78	5.58	0.17 (0.06)	
		31/36	Oral	Letter fluency	Score	44.10	10.00	41.25	14.06	0.23 (0.06)	
		31/36	Oral	Picture naming	Score	55.81	3.03	51.58	5.03	0.99 (0.07)	
	4	30/36	Oral	Category fluency	Score	18.27	4.62	19.78	5.58	-0.29 (0.06)	
		30/36	Oral	Letter fluency	Score	36.36	10.13	41.25	14.06	-0.39 (0.06)	
		30/36	Oral	Picture naming	Score	47.87	6.04	46.11	5.45	0.30 (0.06)	
Soltani et al. (2019)	1	12/12	Oral	Category fluency	Score	10.83	3.48	8.00	2.25	0.93 (0.19)	
		12/12	Oral	Category fluency	Score	16.25	4.67	16.14	4.15	0.02 (0.17)	
		12/12	Oral	Letter fluency	Score	8.00	3.86	6.75	2.59	0.37 (0.17)	
		12/12	Oral	Letter fluency	Score	16.90	4.17	8.41	3.67	2.09 (0.27)	
Stasenko and Gollan (2019)	1	72/72	Oral	Picture naming	Score	65.00	2.30	61.40	3.30	1.26 (0.03)	
Stasenko et al. (2017)	1	79/79	Oral	Picture naming	Score	64.40	2.20	60.70	3.50	1.26 (0.03)	
Sullivan et al. (2018)	1	40/44	Oral	Picture naming	Reaction times	894.80	135.35	965.20	119.40	0.55 (0.05)	R
		40/44	Oral	Picture naming	Percent correct	90.30	4.60	84.80	6.10	1.00 (0.05)	
		40/44	Oral	Picture naming	Reaction times	948.70	135.35	1055.60	135.32	0.78 (0.05)	R
		40/44	Oral	Picture naming	Percent correct	87.60	7.40	74.80	13.60	1.14 (0.06)	
	2	40/22	Oral	Picture naming	Reaction times	894.80	135.35	934.90	83.96	0.33 (0.07)	R
		40/22	Oral	Picture naming	Percent correct	90.30	4.60	82.20	6.90	1.45 (0.09)	
		40/22	Oral	Picture naming	Reaction times	948.70	135.35	1001.80	126.17	0.40 (0.07)	R
		40/22	Oral	Picture naming	Percent correct	87.60	7.40	74.10	14.60	1.27 (0.08)	
	3	52/20	Oral	Picture naming	Reaction times	911.20	113.94	1020.00	166.36	0.83 (0.07)	R
		52/20	Oral	Picture naming	Percent correct	83.40	4.70	73.20	15.80	1.10 (0.08)	
		52/20	Oral	Picture naming	Reaction times	957.30	109.61	1106.70	190.96	1.08 (0.08)	R
		52/20	Oral	Picture naming	Percent correct	83.90	7.30	69.80	17.60	1.26 (0.08)	
	4	52/22	Oral	Picture naming	Reaction times	911.20	113.94	1022.70	161.35	0.85 (0.07)	R
		52/22	Oral	Picture naming	Percent correct	83.40	4.70	76.90	15.20	0.71 (0.07)	
		52/22	Oral	Picture naming	Reaction times	957.30	109.61	1096.40	206.85	0.95 (0.07)	R
		52/22	Oral	Picture naming	Percent correct	83.90	7.30	69.80	16.20	1.31 (0.08)	
Sundaray et al. (2018)	1	19/18	Oral	Letter fluency	Score	48.53	10.93	53.39	10.23	-0.45 (0.11)	
		19/18	Oral	Category fluency	Score	24.30	3.96	24.11	4.79	0.04 (0.11)	

Study	Sample	N (m/b)	Domain	Task	Data type	M	SD	M	SD	Hedges' g	Notes
	2	20/15	Oral	Letter fluency	Score	57.90	19.18	42.53	17.79	0.81 (0.13)	
		20/15	Oral	Category fluency	Score	24.45	5.48	19.13	4.72	1.01 (0.13)	
Taler et al. (2013)	1	32/38	Oral	Picture naming	Score	53.25	3.30	49.43	6.92	0.68 (0.06)	
		32/38	Oral	Letter fluency	Score	40.28	13.53	37.37	10.33	0.24 (0.06)	
		32/38	Oral	Category fluency	Score	24.28	5.82	24.03	6.93	0.04 (0.06)	
Taler et al. (2016)	1	17/18	Oral	Picture naming	Score	53.69	3.23	44.47	8.90	1.33 (0.14)	
		17/18	Oral	Category fluency	Score	26.63	5.85	22.38	5.83	0.71 (0.12)	
		17/18	Oral	Letter fluency	Score	44.13	14.70	34.20	8.73	0.81 (0.12)	
Tao et al. (2015)	1	60/80	Oral	Category fluency	Score	25.00	12.50	20.90	4.60	0.46 (0.03)	
		60/80	Oral	Letter fluency	Score	43.50	11.60	39.30	9.30	0.40 (0.03)	
		60/80	Oral	Picture naming	Score	64.60	2.10	61.90	3.00	1.01 (0.03)	
	2	60/80	Oral	Category fluency	Score	25.00	12.50	23.60	4.60	0.16 (0.03)	
		60/80	Oral	Letter fluency	Score	43.50	11.60	47.90	11.10	-0.39 (0.03)	
		60/80	Oral	Picture naming	Score	64.60	2.10	64.50	2.40	0.04 (0.03)	
Vega-Mendoza et al. (2015)	1	18/16	Oral	Letter fluency	Score	17.78	5.47	17.50	4.55	0.05 (0.12)	
		18/16	Oral	Letter fluency	Score	16.39	3.90	17.44	4.86	-0.23 (0.12)	
		18/16	Oral	Letter fluency	Score	15.50	4.20	17.31	4.30	-0.42 (0.12)	
		18/16	Oral	Category fluency	Score	25.72	5.22	23.94	6.70	0.29 (0.12)	
		18/16	Oral	Category fluency	Score	25.56	5.61	25.69	6.36	-0.02 (0.12)	
		18/16	Oral	Category fluency	Score	21.44	3.70	19.44	4.52	0.48 (0.12)	
	2	18/17	Oral	Letter fluency	Score	17.78	5.47	15.47	4.46	0.45 (0.12)	
		18/17	Oral	Letter fluency	Score	16.39	3.90	15.29	3.06	0.31 (0.12)	
		18/17	Oral	Letter fluency	Score	15.50	4.20	15.59	3.64	-0.02 (0.11)	
		18/17	Oral	Category fluency	Score	25.72	5.22	25.18	5.86	0.10 (0.11)	
		18/17	Oral	Category fluency	Score	25.56	5.61	23.82	4.31	0.34 (0.12)	
		18/17	Oral	Category fluency	Score	21.44	3.70	20.29	3.64	0.31 (0.12)	
Woumans et al. (2015)	1	30/31	Oral	Category fluency	Score	20.20	7.30	12.80	5.40	1.14 (0.08)	
	2	30/28	Oral	Category fluency	Score	20.20	7.30	14.00	4.00	1.03 (0.08)	
Woumans et al. (2019)	1	16/18	Oral	Category fluency	Score	19.06	3.34	19.50	6.06	-0.09 (0.12)	
Yoo and Kaushanskaya (2012)	1	24/21		Picture naming	Standardized scores	117.00	13.18	97.20	12.44	1.51 (0.12)	
Zirnstein et al. (2018)	1	24/28	Oral	Category fluency	Score	44.00	8.00	33.00	6.00	1.55 (0.10)	
Zirnstein et al. (2019)	1	15/12	Oral	Category fluency	Score	45.87	8.30	44.83	12.44	0.10 (0.15)	
Zou et al. (2012)	1	13/14	Oral	Picture naming	Reaction times	784.00	38.00	795.00	107.00	0.13 (0.15)	R
Zou et al. (2012)	1	13/14	Oral	Picture naming	Reaction times	784.00	38.00	795.00	34.00	0.30 (0.15)	R
	1	55/34	Oral	Category fluency	Score	49.20	1.20	41.00	1.70	5.76 (0.24)	O
	1	20/20	Oral	Picture naming	Percent correct	84.70	8.56	74.80	7.92	1.18 (0.12)	
		20/20	Oral	Picture naming	Reaction times	1507.00	243.00	1646.00	308.00	0.49 (0.10)	R
